# Warfarin-Induced Developmental Toxicity: Insights into Embryogenesis, Teratogenicity, and Molecular Pathways

**DOI:** 10.3390/jdb14030034

**Published:** 2026-08-01

**Authors:** Evelyn Magee, Grace Kuhnel, Poongodi Geetha-Loganathan

**Affiliations:** The Department of Biological Sciences, State University of New York Oswego, Oswego, NY 13126, USA; fmagee@oswego.edu (E.M.); gkuhnel@oswego.edu (G.K.)

**Keywords:** warfarin, developmental toxicity, embryogenesis, warfarin fetal syndrome, warfarin signaling, teratogenicity

## Abstract

Warfarin is a coumarin-derived oral anticoagulant widely used for the prevention and treatment of thromboembolic disorders, particularly in patients with mechanical heart valves. The drug exerts its anticoagulant effect by inhibiting vitamin K epoxide reductase, thereby impairing γ-carboxylation of vitamin K-dependent coagulation factors. Despite its clinical efficacy, warfarin therapy is associated with a narrow therapeutic index, substantial interindividual variability in dose response, numerous drug interactions, and significant hemorrhagic risk. Maternal warfarin therapy during pregnancy is strongly associated with fetal warfarin syndrome (FWS), a characteristic pattern of embryopathy resulting from in utero exposure to the drug. This review summarizes current knowledge regarding the physicochemical properties, pharmacological mechanisms, dose variability, toxicity, and developmental effects associated with warfarin exposure. Evidence from human clinical studies and vertebrate animal models is discussed to elucidate conserved developmental and molecular mechanisms underlying warfarin teratogenicity. The review also examines the signaling pathways disrupted by warfarin exposure, highlighting that its teratogenic effects extend beyond anticoagulation to the disruption of vitamin K-dependent developmental signaling. Inhibition of γ-glutamyl carboxylation, together with alterations in Gas6/TAM, PXR, Ras, and Wnt/β-catenin signaling pathways, impairs skeletal, vascular, and neural development, contributing to the characteristic abnormalities of fetal warfarin syndrome. Collectively, this review integrates clinical, molecular, and experimental findings to provide a comprehensive understanding of warfarin-induced developmental toxicity. Current knowledge is insufficient to fully elucidate the complex mechanisms underlying warfarin-induced embryopathy and fetal toxicity. Further investigations are warranted to identify safer anticoagulant regimens during pregnancy and to inform the development of novel therapeutic strategies that minimize fetal risk while maintaining maternal anticoagulation.

## 1. Introduction

**Figure 1:**
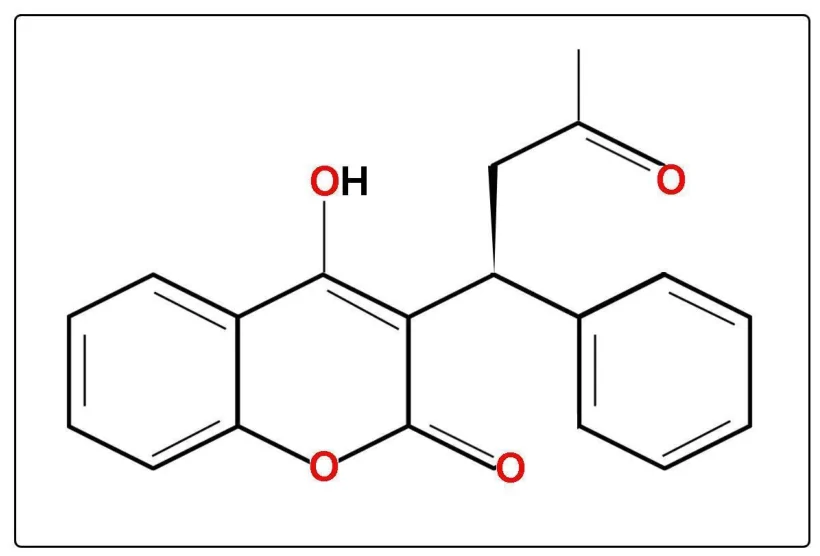
**Structure of S-Warfarin.** An anticoagulant drug, (S)-warfarin sodium is an organic sodium salt having 2-oxo-3-[(1S)-3-oxo-1-phenylbutyl]-2*H*-1-benzopyran-4-olate. It is an enantiomer of (R)-warfarin. Reference: PubChem CID 54688261.

Warfarin is a synthetic 4-hydroxycoumarin derivative belonging to the coumarin class of anticoagulants and is widely used for the prevention and treatment of thromboembolic disorders. Chemically, warfarin (C_19_H_16_O_4_; molecular weight 308.33 g/mol) is administered clinically as a racemic mixture of R- and S-enantiomers, with the S-enantiomer exhibiting approximately 3–5 times greater anticoagulant potency than the R-enantiomer due to differences in hepatic metabolism and affinity for vitamin K epoxide reductase (VKOR) (Figure 1) [1]. Owing to its poor aqueous solubility, warfarin is commonly formulated as the more water-soluble sodium salt to improve oral bioavailability.

Warfarin is a weak acid (pKa 5.0–5.2) and is extensively bound to plasma albumin (>99%), with hepatic metabolism occurring primarily through cytochrome P450 enzymes, particularly CYP2C9 for S-warfarin [1]. These pharmacokinetic characteristics (Table 1) influence systemic drug exposure, maternal clearance, and fetal exposure during pregnancy. Pharmacological properties of selected warfarin analogs reported in experimental studies are seen in Table S1. Importantly, despite its high plasma protein binding, warfarin readily crosses the placenta, exposing the developing embryo and fetus to pharmacologically active concentrations, which underlies its well-established teratogenic potential.

Warfarin and related coumarin anticoagulants, including phenprocoumon and acenocoumarol, share a common 4-hydroxycoumarin scaffold and exert their anticoagulant activity by inhibiting the vitamin K cycle. Specifically, they inhibit vitamin K epoxide reductase (VKOR), preventing regeneration of vitamin K hydroquinone, the essential cofactor required for γ-glutamyl carboxylation and activation of vitamin K-dependent proteins involved in coagulation and skeletal development [2,3]. These pharmacological properties form the mechanistic basis for the developmental abnormalities associated with fetal warfarin exposure.

The anticoagulant properties of warfarin were first recognized following investigations into fatal hemorrhagic disease in cattle that consumed spoiled sweet clover in the early twentieth century [3]. After its commercial introduction as a rodenticide in 1948, warfarin was subsequently adapted for clinical use in the 1950s and gained widespread medical acceptance following its administration to President Dwight Eisenhower after his 1955 myocardial infarction [4]. Since then, warfarin has remained a cornerstone therapy for the prevention and management of thromboembolic disorders, including deep vein thrombosis (DVT), pulmonary embolism, and cardioembolic stroke [5]. Although several direct oral anticoagulants have emerged as alternatives to warfarin, specific clinical scenarios, particularly anticoagulation in patients with mechanical heart valves, continue to require warfarin due to the limited efficacy or safety of current alternatives [6].

Despite its therapeutic importance, warfarin is a well-established teratogen associated with dose-dependent embryofetal toxicity and a spectrum of developmental abnormalities collectively termed warfarin embryopathy [7]. Warfarin readily crosses the placenta and can enter the fetal circulation, thereby exposing the developing fetus to its pharmacological and teratogenic effects. Consequently, anticoagulation management during pregnancy presents a significant clinical challenge in balancing maternal thromboembolic risk against fetal developmental toxicity. Here, we aim to combine all information on the properties, mechanism of action, and signaling mechanisms underlying warfarin toxicity, as well as its teratogenic effects during development.

### Literature Search and Study Selection

The search for original articles was performed using databases, including Scopus, PubMed, Google Scholar, and ScienceDirect, with MeSH terms such as *warfarin, anticoagulant therapy, warfarin embryopathy, fetal warfarin syndrome, warfarin teratogenicity, and effects of warfarin during development.* The final comprehensive database search was completed on 14 July 2026, and additional relevant publications identified during manuscript revision were evaluated individually and incorporated where appropriate prior to final submission. Database-specific search strategies were adapted to the requirements of each database. Representative database-specific search strings included Google Scholar queries such as “Warfarin Teratogenesis,” “Vitamin K uptake,” “Vitamin K metabolism,” and “Warfarin zebrafish.” Equivalent Boolean combinations of these keywords were adapted for PubMed, Scopus, and ScienceDirect according to each database’s search syntax.

Web sources such as the National Institutes of Health (NIH), the World Health Organization (WHO), and NCBI-PubChem were also referred to obtain information on warfarin. To ensure a comprehensive literature search, AI-assisted tools including Litmaps (2024), ChatGPT (GTP 5.5) and Gemini (version 3) were used to identify relevant information and sources that may not have been captured through conventional databases, including some of the research publications, selected webpages like National Center for Biotechnology Information (Pubchem) and fda.gov (FDA drug sheet). All information identified through AI tools was independently verified against original, authoritative sources before being incorporated into the manuscript. The AI tools were used solely to support literature discovery and learning and were not used to generate or write the manuscript.

Peer-reviewed studies published in English, including original research studies, clinical trials, reviews, and case reports, were considered for inclusion. Relevant studies were selected through title, abstract, and full-text screening, and additional references were identified from the bibliographies of selected articles. The overall reference list (*n* = 110) was assembled through this structured search process to provide comprehensive coverage of warfarin pharmacology, molecular mechanisms, developmental biology, experimental animal models, clinical applications, teratogenicity, and alternative anticoagulant therapies. In contrast, the formal screening process described below was applied specifically to published clinical case reports included in the phenotype analysis.

This review was conducted as a narrative literature review with a structured search strategy rather than a formal systematic or scoping review. The literature search included publications from 1970 to 2026, with the final search completed prior to manuscript preparation. A total of 28 case studies addressing the developmental effects of warfarin exposure were identified and screened for relevance. Of these, 25 studies met the inclusion criteria, while 3 studies were excluded. Studies were eligible for inclusion if they reported teratogenic or developmental effects specifically associated with documented maternal warfarin exposure, including information on warfarin administration and, where available, dosage. Studies describing vitamin K deficiency phenotypes without confirmed warfarin exposure, as well as those investigating only other coumarin anticoagulants (e.g., phenprocoumon or acenocoumarol), were excluded from the primary analysis.

Duplicate bibliographic records retrieved from multiple databases were identified by comparing article title, authors, publication year, and journal information and were removed prior to full-text evaluation. Because the objective of this review was to summarize and discuss the reported spectrum of developmental abnormalities rather than perform a quantitative synthesis or estimate phenotype prevalence, duplicate clinical case reports describing the same patient(s) were not formally assessed, and the phenotype summary table is intended as a descriptive catalogue of reported abnormalities and their original literature sources rather than a pooled dataset.

The references included in this review span publications from 1970 to 2026 (*n* = 110 references), with a median publication year of 2008, reflecting both the historical development of the field and recent advances in understanding the molecular mechanisms underlying warfarin teratogenicity (Table 2). This manuscript is a narrative review and does not follow a formal systematic review protocol. The retrieved literature was thus critically evaluated and synthesized to provide a comprehensive overview of warfarin pharmacology, clinical applications, adverse effects, teratogenicity, and alternative anticoagulant therapies.

## 2. Pharmacological Mechanism of Warfarin

Warfarin exerts its anticoagulant effect by inhibiting the vitamin K cycle, thereby preventing the post-translational activation of vitamin K-dependent proteins (VKDPs). Vitamin K exists as phylloquinone (vitamin K_1_) and menaquinones (vitamin K_2_). Dietary vitamin K_1_ is absorbed in the proximal small intestine through transporters including scavenger receptor class B type 1 (SR-BI), cluster of differentiation 36 (CD36), and Niemann–Pick C1-like 1 (NPC1L1) [8]. A portion is converted to menaquinone-4 by UBIAD1 in peripheral tissues, where it functions in both γ-carboxylation and cellular signaling [8,9].

Vitamin K hydroquinone serves as an essential cofactor for γ-glutamyl carboxylase (GGCX), which catalyzes γ-carboxylation of VKDPs to produce biologically active proteins [10]. During this process, vitamin K is oxidized to vitamin K epoxide and subsequently recycled by vitamin K epoxide reductase (VKOR). Warfarin competitively inhibits VKOR, depleting active vitamin K and impairing γ-carboxylation of VKDPs. As a result, activation of coagulation factors II, VII, IX, and X, together with proteins C and S, is reduced. In addition to coagulation, VKDPs are involved in bone mineralization, skeletal development, and cellular signaling [11].

Warfarin is rapidly absorbed after oral administration, reaching peak plasma concentrations within 0.3–4 h [3]. More than 99% is bound to plasma albumin, while hepatic uptake may involve organic anion transporter 2 (OAT2) and breast cancer resistance protein (BCRP) [12,13]. Following distribution, warfarin accumulates primarily in hepatic and renal tissues [14]. The drug is administered as a racemic mixture of R- and S-warfarin, with the S-enantiomer exhibiting greater anticoagulant potency. Hepatic metabolism is stereoselective, with CYP2C9 primarily metabolizing S-warfarin to hydroxywarfarin metabolites, whereas R-warfarin is metabolized more slowly by other cytochrome P450 enzymes [3]. Metabolites are excreted mainly in urine, and S-warfarin is eliminated approximately twice as rapidly as R-warfarin. Variants in CYP2C9, VKORC1, and CYP4F2 contribute significantly to interindividual differences in warfarin metabolism, sensitivity, and dose requirements [5,15,16].

Warfarin binds reversibly within the VKOR active site through hydrophobic and aromatic interactions, with residues Y139, Y25, and A26 playing critical roles in ligand binding [17]. Mutations in these residues reduce warfarin affinity and confer resistance [17,18]. Inhibition of VKOR disrupts vitamin K recycling and VKDP activation, providing the molecular basis for both the therapeutic anticoagulant action of warfarin and the developmental defects associated with fetal warfarin syndrome.

## 3. Warfarin Dose Variability, Toxicity, and Clinical Complications

Warfarin therapy requires meticulous dose individualization owing to its narrow therapeutic index, marked interindividual variability, and complex pharmacokinetic and pharmacodynamic profile. Therapeutic dosing is influenced by numerous clinical variables, including age, body mass, hepatic function, dietary vitamin K intake, comorbidities, and concomitant pharmacotherapy. Warfarin is administered as a racemic mixture comprising the pharmacologically distinct S- and R-enantiomers, which exhibit elimination half-lives of approximately 32 h and 43 h, respectively [3]. Owing to considerable variability in anticoagulant response, therapeutic monitoring is achieved through serial assessment of the prothrombin time, a blood test that measures how long it takes blood to clot through the extrinsic and common coagulation pathways and is standardized as the international normalized ratio (INR), which remains the cornerstone of dose titration and anticoagulation management.

Genetic determinants contribute substantially to variability in warfarin sensitivity and maintenance dose requirements. Polymorphisms in *CYP2C9*, the principal enzyme responsible for oxidative metabolism of S-warfarin, reduce metabolic clearance and prolong systemic exposure to the active enantiomer. In particular, the CYP2C9*3 allele has consistently been associated with lower maintenance dose requirements, delayed attainment of stable anticoagulation, and an increased risk of over-anticoagulation during treatment initiation [15]. Variants in *VKORC1*, which encodes vitamin K epoxide reductase complex subunit 1, exert additional pharmacodynamic effects by altering sensitivity to VKOR inhibition, thereby representing a major determinant of dose variability across populations [16]. Polymorphisms in *CYP4F2*, which participate in vitamin K oxidation, have also been implicated in modulating dose requirements, although their contribution appears to be comparatively modest [5]. Consequently, incorporation of pharmacogenetic data into dosing algorithms has emerged as an important strategy for optimizing anticoagulation therapy and minimizing adverse outcomes.

Hemorrhage remains the most clinically significant adverse effect associated with warfarin therapy and represents the principal limitation to its long-term use [19,20]. Bleeding complications may range from minor mucocutaneous manifestations to severe or fatal hemorrhage involving the gastrointestinal tract, intracranial compartment, or other major organ systems [21]. The risk of hemorrhage is influenced by excessive anticoagulation, advanced age, interacting medications, hepatic dysfunction, and comorbid disease states [3]. In addition to bleeding complications, warfarin therapy has been associated with systemic atheroembolism and cholesterol microembolization syndromes.

A broad spectrum of non-hemorrhagic adverse effects has also been described. Immunologic and hypersensitivity manifestations include allergic reactions and vasculitic phenomena, while hepatobiliary complications encompass hepatitis and elevations in hepatic transaminases [22]. Gastrointestinal adverse effects may include nausea, vomiting, diarrhea, abdominal pain, bloating, flatulence, and dysgeusia. Dermatologic complications, such as rash, dermatitis, pruritus, and alopecia, have also been reported. Less frequently, respiratory complications, including tracheal and tracheobronchial calcification, may occur, together with generalized constitutional symptoms such as chills [22]. Among the most severe non-hemorrhagic complications is warfarin-induced skin necrosis, a rare but potentially life-threatening condition associated with substantial morbidity and mortality [23,24]. This complication most commonly develops in the initial days of therapy and is believed to arise from a transient hypercoagulable state secondary to the rapid depletion of the endogenous anticoagulant proteins C and S relative to procoagulant factors. Clinically, affected patients initially develop painful erythematous lesions that may rapidly progress to purpura, hemorrhagic bullae, and full-thickness cutaneous necrosis, particularly within adipose-rich anatomical regions such as the breasts, thighs, buttocks, and abdomen. Early recognition and prompt discontinuation of warfarin are critical to limiting tissue destruction and improving clinical outcomes.

## 4. Phenotypic Spectrum of Fetal Warfarin Syndrome in Humans

Warfarin exposure during pregnancy is associated with significant embryotoxic and fetotoxic risk. The severity of fetal complications demonstrates a dose-dependent relationship, with higher maternal doses correlating with increased incidence of spontaneous abortion, stillbirth, and warfarin embryopathy [19]. Maternal doses exceeding 5 mg/day were associated with substantially greater risk of adverse fetal outcomes. The teratogenic effects of warfarin are primarily attributed to disruption of vitamin K-dependent γ-carboxylation pathways essential for normal skeletal and connective tissue development during embryogenesis.

Warfarin remains one of the few anticoagulants considered effective for preventing thromboembolism in pregnant patients with mechanical heart valves, despite its well-established teratogenic potential [19,25]. Maternal warfarin dosage may influence the likelihood of adverse fetal outcomes. Pregnant patients receiving warfarin doses greater than 5 mg daily have demonstrated increased rates of fetal complications, including spontaneous abortion, compared with patients maintained on doses of 5 mg daily or less. Most documented cases of warfarin embryopathy involve maternal doses between 5 and 7.5 mg daily, although many do not specify a dosage. Importantly, the most common abnormalities—including nasal hypoplasia, saddle nose deformity, epiphyseal stippling, and distal phalangeal hypoplasia—have been reported following both low- and high-dose warfarin exposure. Fetal exposure to warfarin during pregnancy can result in a spectrum of congenital abnormalities collectively referred to as warfarin embryopathy (WE) or fetal warfarin syndrome (FWS) [26,27,28]. The resulting phenotypes are highly variable and depend on the timing, duration, and dosage of exposure during gestation. The risk of classic warfarin embryopathy is greatest when exposure occurs during the first trimester, particularly between the sixth and ninth weeks of gestation, when organogenesis and cartilage development are highly active [26,29,30]. However, adverse fetal outcomes may also occur following second- and third-trimester exposure, resulting in severe neurological injury, growth restriction, and fetal loss, indicating that teratogenicity is not restricted to early gestation.

**Figure 2:**
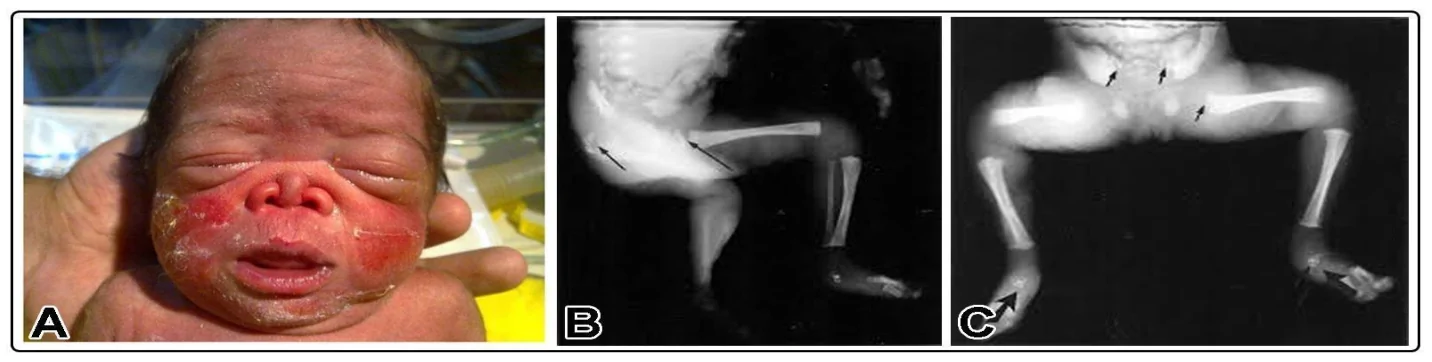
**Photographic** (**A**) **and radiographic** (**B**,**C**) **representation of Warfarin Embryopathy.** (**A**) Preterm newborn at 33 weeks of pregnancy delivered a male fetus with nasal hypoplasia characterized by a depressed nasal bridge and flat upturned nose (taken from Gupta et al., 2010) [43]. (**B**,**C**) Aborted fetus following termination of pregnancy at 23 2/7 weeks’ gestation, showing bilateral calcific stippling of the proximal femoral epiphyses (**B**, **arrows**), the iliac wings, and the tarsal bones (**C**, **arrows**) (taken from Chan et al., 2003) [31].

Warfarin embryopathy demonstrates considerable phenotypic variability and can affect the craniofacial, musculoskeletal, cardiopulmonary, and central nervous systems. The most characteristic feature is nasal hypoplasia, typically presenting with a depressed nasal bridge, a short upturned nose, and underdevelopment of the nasal cartilage (Figure 2A) [19,26,29,30,31,32,33,34,35,36,37,38,39,40,41,42]. Additional nasal abnormalities commonly reported alongside nasal hypoplasia include a flattened or absent nasal bridge, deep ala nasi grooves, anteverted nostrils, nasal stenosis, absent nasal bones, choanal atresia, and respiratory complications secondary to laryngomalacia or tracheomalacia [29,31,35,43]. Other craniofacial findings include micrognathia, cleft lip or palate, frontal bossing, hypertelorism, and facial asymmetry [31,32,43]. Some infants also present with choanal stenosis or airway abnormalities, including laryngomalacia and tracheomalacia, which may contribute to neonatal respiratory distress (Table 3 and Table S2).

Another hallmark of warfarin embryopathy is epiphyseal stippling, also known as chondrodysplasia punctata, which results from abnormal calcification in developing cartilage. Epiphyseal stippling may affect multiple skeletal regions, including the ankles, hips, tarsals, patellae, phalanges, talus, and most frequently within the humerus, vertebral column, femur, and calcaneus (Figure 2B,C) [26,27,30,31,32,34,35,36,38,39,41,42,43,44]. Infants with nasal hypoplasia frequently demonstrate concomitant epiphyseal stippling, reflecting the high prevalence of these two defining phenotypes within fetal warfarin syndrome [29,30,31,32,35,36,38]. Although stippling may diminish with age, affected individuals frequently exhibit persistent orthopedic abnormalities, including limb shortening, scoliosis, joint deformities, and abnormal gait.

**Table 3:** Craniofacial Phenotypes Reported Following Prenatal Warfarin Exposure in Humans.

Phenotype Category	Representative Clinical Findings	Representative Maternal Exposure	Typical Gestational Exposure	Evidence Strength	Representative References
Nasal hypoplasia and facial hypoplasia	Nasal, maxillary, mandibular, and midfacial hypoplasia; short/small nose; flattened face	Therapeutic doses (3–12.5 mg/day), most commonly 5–10 mg/day	Most frequently associated with first-trimester exposure (weeks 6–12), although continued exposure throughout pregnancy has also been reported	Multiple case reports and case series	[19,27,29,30,31,32,34,35,36,37,38,39,40,41,42,43,45,46]
Nasal structural abnormalities	Saddle nose, absent nasal bones, nasal stenosis, deep ala nasi groove, anteverted nostrils, choanal atresia	3–10 mg/day	First-trimester exposure or continued exposure during pregnancy	Multiple case reports	[19,27,29,31,35,36,38,39,40,41,42,43,44,47]
Oral and airway abnormalities	Microstomia, laryngomalacia with tracheomalacia	Mainly therapeutic doses (~5 mg/day)	Throughout pregnancy or prolonged exposure	Isolated case reports	[31,36,42]
Orbital and cranial abnormalities	Hypertelorism, macrocephaly, occipital flattening	5–10 mg/day	Throughout pregnancy	Case reports	[29,35,41,44,45]
Auricular abnormalities	Auricular fold hypoplasia, pinnae hypoplasia	Not consistently reported	Not consistently reported	Isolated case reports	[31]

Note: Representative exposure ranges are summarized from published reports; individual doses varied among patients.

Skeletal abnormalities beyond epiphyseal stippling are also frequently observed. Distal phalangeal hypoplasia, limb shortening, and generalized growth restriction have been repeatedly documented in affected infants. Although these findings occur less consistently than nasal hypoplasia or stippled epiphyses, they remain important diagnostic indicators of prenatal warfarin exposure. Hypoplastic distal phalanges, brachydactyly, shortened extremities, and nail abnormalities have also been described in infants with FWS. Additional musculoskeletal findings include vertebral anomalies, pectus deformities, hip dysplasia, and delayed skeletal maturation. In severe cases, generalized growth restriction and low birth weight may accompany these structural abnormalities, suggesting broader disruption of fetal growth and connective tissue development (Table 4 and Table S3) [19,26,27,29,30,32,35,36,39,45,46].

Warfarin exposure also causes cardiovascular (Table 5 and Table S4) and ophthalmologic (Table 6 and Table S5) abnormalities during development. Although less consistently observed than skeletal and craniofacial findings, congenital heart defects including septal defects and outflow tract abnormalities have occasionally been reported following prenatal warfarin exposure [37,42]. Ocular phenotypes such as microphthalmia, optic nerve abnormalities, cataracts, and visual impairment have also been reported [26,37]. Further, in a few cases, hearing disability and genitourinary abnormalities have been documented [27,47].

Neurological abnormalities associated with prenatal warfarin exposure are often the most clinically significant outcomes compared to the classic skeletal and craniofacial manifestations of warfarin embryopathy because of their potential to cause lifelong disability [26,47,50]. CNS anomalies are more likely to develop following exposure during the second and third trimesters of pregnancy that result from fetal hemorrhage, subsequent scarring, and secondary impairment of normal brain development (Table 7 and Table S6) [26,51]. Reported CNS findings include intracranial hemorrhage, cerebral cysts, ischemic lesions, hydrocephalus, calcified brain regions, and ventriculomegaly [26,30,31,42,47,48,50,51,52]. Hydrocephalus, agenesis of the corpus callosum, cerebral hypoplasia, cortical atrophy, seizures, and intellectual disability have also been documented in multiple reports [31,32,33,35,37,46,47,50,51]. Collectively, these findings demonstrate that fetal warfarin exposure results in a broad and variable constellation of developmental abnormalities. While craniofacial and skeletal defects are the most recognizable manifestations of warfarin embryopathy, CNS involvement contributes substantially to long-term morbidity and developmental impairments, including speech, motor coordination, and learning. The diversity and severity of these phenotypes emphasize the importance of careful anticoagulant management during pregnancy and continued investigation into safer therapeutic alternatives for patients requiring long-term anticoagulation.

Prenatal warfarin exposure has also been associated with a range of additional abnormalities beyond the classic manifestations of fetal warfarin syndrome, including impaired fetal growth, hearing deficits, developmental delay, nail hypoplasia, and coagulation disturbances (Table 8 and Table S7). These findings suggest that warfarin embryopathy may affect multiple organ systems and developmental processes.

FWS is a complex teratogenic disorder with substantial phenotypic variability. Although typical phenotypes presented include nasal hypoplasia and epiphyseal stippling, warfarin exposure has been associated with a broader range of craniofacial, skeletal, neurological, cardiovascular, and respiratory abnormalities. Understanding the pathogenic basis of warfarin-induced embryopathy may aid in improving prenatal risk assessment and the development of safer anticoagulant therapies.

### Strengths and Limitations of the Clinical Evidence

As described above, warfarin embryopathy is one of the well-diagnosed drug-induced teratogenic syndromes, but the available clinical evidence is mostly based on observational studies from pregnancy series, and individual case reports. In this section, the certainty of the evidence is evaluated across the reported manifestations to interpret the full phenotypic spectrum.

The evidence supporting a dose-dependent relationship is less robust as the frequently cited 5 mg/day threshold is based primarily on the study by Vitale et al. (1999) [19] of pregnant women with mechanical heart valves. While this study demonstrated fewer fetal complications among women receiving ≤5 mg/day, the analysis was limited by a relatively small cohort, heterogeneous clinical management, and restriction to patients with mechanical prosthetic valves. These findings, therefore, could not be generalizable to all pregnant women requiring anticoagulation. Also, fetal warfarin syndrome is reported following maternal doses of 5 mg/day or less [41,42] indicating that teratogenic risk cannot be excluded at lower doses. Maternal dose alone is also an indicator of fetal exposure because anticoagulant response varies with CYP2C9 and VKORC1 polymorphisms, dietary vitamin K intake, concurrent medications, and maternal pharmacokinetics. Therefore, current evidence supports a graded increase in fetal risk with higher doses but does not define a clear safety threshold. The mechanistic work of Pauli et al. (1987) [27], demonstrating similarities between warfarin embryopathy and inherited deficiencies of vitamin K-dependent proteins, further strengthens this conclusion by providing biological evidence that complements the clinical observations. Potential confounding factors should also be acknowledged. Women receiving warfarin during pregnancy commonly have mechanical heart valves or severe thromboembolic disorders, conditions that independently increase the risks of miscarriage, placental dysfunction, fetal growth restriction, and adverse pregnancy outcomes. Consequently, fetal loss and some non-specific complications cannot always be attributed solely to warfarin exposure.

In contrast, the evidence is strong for the association between first-trimester warfarin exposure and the characteristic skeletal and craniofacial abnormalities of fetal warfarin syndrome. Since the initial descriptions by multiple independent reports have consistently identified nasal hypoplasia, depressed nasal bridge, stippled epiphyses (chondrodysplasia punctata), and distal phalangeal hypoplasia in infants exposed to warfarin during early organogenesis [26,33,45]. These recurring findings have been reproduced over several decades in different clinical settings and populations, suggesting that they represent the core phenotype of warfarin embryopathy rather than isolated observations. Several methodological limitations reduce the certainty of the available evidence. Some published reports lack detailed information on maternal warfarin dosage, gestational timing and duration of exposure, concomitant medications, and maternal comorbidities, limiting accurate assessment of exposure-response relationships. Because fetal susceptibility varies across gestation, incomplete exposure data hinder identification of critical windows of teratogenicity. Moreover, many landmark studies were published before the widespread use of high-resolution prenatal ultrasonography, fetal magnetic resonance imaging, fetal echocardiography, and molecular genetic testing. As a result, some congenital anomalies may have been incompletely characterized or misclassified according to current diagnostic standards.

Craniofacial and skeletal abnormalities have been documented repeatedly across numerous independent studies and are therefore regarded as well-established manifestations of fetal warfarin syndrome. In comparison, cardiovascular, ophthalmologic, respiratory, hearing, and genitourinary abnormalities are reported much less frequently and are often described in isolated case reports or small case series. Although these abnormalities may represent true consequences of prenatal warfarin exposure, the available evidence is insufficient to determine whether they are consistently attributable to warfarin or reflect coincidental congenital anomalies occurring in a population already at increased obstetric risk. The quality of evidence also varies according to the organ system involved and these findings should be interpreted as infrequent or possible manifestations rather than defining features of the syndrome. Similarly, the neurological complications of prenatal warfarin exposure require similar consideration. Reports of fetal intracranial hemorrhage are relatively consistent across the literature [50,51,52] and are supported by warfarin’s known anticoagulant mechanism. However, secondary neurological outcomes such as hydrocephalus, cortical atrophy, seizures, developmental delay, and intellectual disability are considerably more heterogeneous. In many cases, these abnormalities appear to result from hemorrhagic injury sustained during fetal life rather than direct interference with embryonic neural patterning. Consequently, although neurological morbidity is a recognized consequence of prenatal warfarin exposure, the underlying pathogenic mechanisms likely differ from those responsible for the classic skeletal phenotype and require further investigation.

Clinical guidelines published by the American Heart Association/American College of Cardiology [21], the American College of Chest Physicians [20], the FDA prescribing information, and subsequent reviews by Gibson and Powrie (2009) [25] consistently recognize warfarin as a proven human teratogen. However, these recommendations are based primarily on a synthesis of the existing observational literature rather than new prospective clinical evidence. Therefore, they reinforce the clinical consensus regarding teratogenic risk but do not overcome the inherent methodological limitations of the underlying studies. Overall, the available evidence strongly supports a causal association between first-trimester warfarin exposure and the characteristic craniofacial and skeletal features of fetal warfarin syndrome. Neurological complications following later gestational exposure are also well documented, whereas evidence for cardiovascular, ophthalmologic, respiratory, and genitourinary abnormalities remains limited and is based largely on isolated case reports. Prospective multicenter pregnancy registries with standardized exposure and outcome reporting are needed to better define the full phenotypic spectrum and quantify fetal risk.

## 5. Insights from Animal Models of Fetal Warfarin Syndrome

Vertebrate animal models provide valuable systems for studying the molecular and phenotypic mechanisms underlying fetal warfarin syndrome (FWS) due to rapid embryonic development, high fecundity, and conserved genetic pathways between zebrafish and humans. Several signaling pathways involved in coagulation and vitamin K metabolism, which are required for skeletal and vascular development, are evolutionarily conserved between vertebrate model organisms and humans, making them useful for developmental toxicology studies [53,54].

### 5.1. Zebrafish

Experimental exposure of zebrafish (*Danio rerio*) embryos to warfarin has demonstrated dose-dependent, developmental-stage-specific effects during embryogenesis and organ development. Zebrafish embryos exposed to warfarin concentrations ranging from 62.5 to 1500 μM showed a relationship between increasing warfarin concentration and the severity of teratogenic and lethal outcomes [55,56]. At the highest concentration, 1500 μM warfarin exhibited 92% mortality rate within 2–3 days post-fertilization (dpf) in embryos, and developmental defects were detectable within 1 dpf, indicating accelerated toxicity. The embryos exposed to a higher concentration of warfarin showed severe developmental abnormalities such as absent cardiac activity and coagulation prior to death. Survived embryos demonstrated delayed early development at 1 dpf followed by severe morphological abnormalities during later stages. Morphological phenotypes include defects of the head, eyes, sacculi and otoliths, notochord, tail, and tail tip, as well as scoliosis, yolk deformities, and generalized growth impairment. Notochord abnormalities were dose-dependent, appearing as isolated lesions in lower-dose groups but progressing to extensive structural disintegration at higher concentrations [55,56]. At lower concentrations (62.5–250 μM), teratogenic abnormalities generally became apparent at approximately 3 dpf, whereas embryos exposed to 500–1000 μM exhibited abnormalities as early as 2 dpf. Toxic effects of warfarin at concentrations of 5, 25, and 125 mg/L during embryonic development (1 h to 2.5 dpf) and endotrophic development (2.5–5 dpf) were recorded to manifest reduced survival rate, reduced body size, and persistent growth impairment with hemorrhagic events [57]. Warfarin exposure also induced substantial cardiovascular and skeletal abnormalities. Cardiac edema occurred more frequently during embryonic-stage exposure, whereas swim bladder reduction was observed at the highest concentration (125 mg/L) regardless of developmental stage. Embryos exposed to warfarin frequently exhibit cardiac edema, reduced heart rate, altered cardiac looping, and circulatory defects. Such observations may help explain the congenital cardiovascular abnormalities occasionally reported in human fetal warfarin syndrome. Warfarin exposure at 20 μM from 4 to 96 h post-fertilization (hpf) in zebrafish embryos has significantly reduced cardiac cone area and heart tube size, indicating impaired early cardiac development [58]. Although heart tube formation still occurred, affected embryos exhibited incomplete and defective cardiac looping, reduced chamber size, and impaired rhythmic contraction. Structural cardiac abnormalities included a markedly smaller ventricle, atrial dilation, and defective endocardial cushion formation. Minor looping abnormalities were also observed, suggesting disruption of normal cardiac patterning and morphogenesis. This study identified a broad critical exposure window extending from 4 to 72 hpf, indicating that warfarin-induced cardiotoxicity affects multiple stages of zebrafish cardiac development.

Cartilage development was also disrupted by warfarin treatment. Embryos exposed to the highest concentration (125 mg/L) exhibited reduced cartilage growth plate size in the ethmoid, Meckel’s, and ceratohyal cartilages, with embryonic-stage exposure again producing more severe defects. These defects are thought to arise from impaired neural crest cell differentiation and abnormal extracellular matrix mineralization. Given that craniofacial anomalies such as nasal hypoplasia represent hallmark features of fetal warfarin syndrome in humans, zebrafish provide an experimentally accessible model for investigating the developmental origins of these abnormalities. Skeletal analyses demonstrated impaired mineralization of several dermal and endochondral skeletal structures, including the cleithrum, parasphenoid, basioccipital, and ceratobranchial bones. Exposure during endotrophic development primarily delayed mineralization of the parasphenoid, whereas embryonic-stage exposure resulted in a near-complete absence of mineralization in the cleithrum and parasphenoid at 5 and 7 dpf. Severe anti-mineralogenic effects were therefore most pronounced following early developmental exposure. Mineralization of vertebral centra was also markedly reduced, particularly in the caudal and caudal fin vertebrae. Additional histological abnormalities included hepatocellular shrinkage and thinning of retinal layers, especially following embryonic-stage exposure. Significant reductions in the ganglion cell layer, inner plexiform layer, and inner nuclear layer were observed at 7 dpf. Collectively, these findings demonstrated that warfarin exposure increased mortality, impaired growth, induced hemorrhage, shortened lifespan, disrupted skeletal mineralization, and altered cartilage development, with embryos exposed during early embryogenesis showing the most severe phenotypes (Table 9) [57].

The zebrafish model has emerged as a valuable experimental system for investigating warfarin-induced developmental toxicity due to its unique biological characteristics, including external fertilization, transparent embryos, rapid development, and the capacity for high-throughput analysis of developmental effects across precisely defined stages. The established zebrafish developmental atlas allows researchers to associate specific exposure periods with corresponding morphological and physiological abnormalities [54]. Studies investigating warfarin exposure in zebrafish have reported a range of adverse developmental outcomes, including concentration- and stage-dependent mortality, hemorrhagic events, reduced growth, axial skeletal abnormalities, craniofacial malformations, cardiac defects, and impaired mineralization processes [55,56,57]. Several of these abnormalities parallel clinical manifestations observed in fetal warfarin syndrome, particularly defects affecting craniofacial structures, skeletal development, cardiovascular formation, and overall fetal growth [57]. Beyond reproducing phenotypic features associated with human warfarin embryopathy, zebrafish models have contributed to understanding the molecular mechanisms responsible for these developmental defects. Granadeiro et al. (2019) [57] demonstrated that warfarin exposure disrupts cartilage differentiation, decreases mineral deposition in both dermal and endochondral skeletal tissues, and affects vascular and cardiac development, highlighting the importance of vitamin K-dependent pathways during embryogenesis. Furthermore, experimental manipulation of developmental signaling cascades, particularly Wnt signaling, has revealed that altered pathway activity can modify warfarin-induced craniofacial abnormalities, suggesting that secondary developmental networks contribute to the teratogenic response [55,57]. These findings establish zebrafish as a powerful platform for elucidating mechanisms of warfarin toxicity and for evaluating potential protective or therapeutic interventions. Despite these advantages, interpretation of zebrafish data requires consideration of important biological differences from mammalian systems. Variations in embryonic structure, xenobiotic metabolism, and nutrient uptake mechanisms may influence the response to warfarin exposure. In addition, the absence of a mammalian placenta and maternal-fetal circulation prevents direct assessment of maternal pharmacokinetic factors, placental transport, and fetal exposure dynamics. Therefore, while zebrafish provide valuable mechanistic insights into warfarin-induced developmental abnormalities, findings must be integrated with mammalian and clinical studies to accurately define their relevance to human pregnancy.

### 5.2. Chicken

The chick embryo is particularly useful for teratogenicity studies because embryonic development occurs externally, allowing direct manipulation and observation of skeletal and vascular development during defined developmental stages. The effects of dietary warfarin exposure in Leghorn and broiler chicks fed diets containing 25, 50, or 75 mg warfarin/kg body weight were reported [60]. Hemorrhagic lesions increased in severity with increasing warfarin dosage. Large subcutaneous hemorrhages were particularly evident in the wings of both breeds receiving the highest warfarin concentration. Intramuscular hemorrhages were also observed in several muscle groups, including the major and minor pectoralis, biceps femoris, and sartorius muscles, with occasional hemorrhage extending into the intraperitoneal region. Higher warfarin concentrations were additionally associated with increased mortality rates. Both Leghorn and broiler chicks fed warfarin-containing diets demonstrated significant reductions in feed consumption and body weight gain throughout most observation periods compared with controls (Table 10). Prothrombin times were also significantly prolonged in warfarin-treated birds, confirming the anticoagulant effects of warfarin exposure [60].

The chicken embryo is a useful model for studying developmental toxicity because of its external development, accessibility, and suitability for visualizing and manipulating vascular, skeletal, and craniofacial processes. Warfarin studies in Leghorn and broiler chickens have demonstrated dose-dependent hemorrhage, reduced growth, increased mortality, and prolonged prothrombin time, confirming disruption of vitamin K-dependent coagulation pathways [60]. However, the relevance of chicken models to fetal warfarin syndrome is limited because many studies focus on postnatal or systemic exposure rather than embryonic exposure during organogenesis. In addition, differences in avian and mammalian development, including the absence of placental interactions and distinct skeletal maturation processes, limit direct extrapolation to human pregnancy. Nevertheless, the chicken embryo remains valuable for investigating developmental responses and mechanisms of warfarin toxicity.

### 5.3. Rats

Rodent models have provided important insight into the developmental and skeletal effects of prenatal warfarin exposure and have helped clarify the mechanisms underlying fetal warfarin syndrome [61]. The effects of warfarin exposure in pregnant Sprague–Dawley rats were studied by administering 100 mg/kg warfarin orally each day in combination with intramuscular injections of 10 mg/kg vitamin K1. When treatment occurred during gestational days 1–12, no major maternal or fetal abnormalities were observed apart from fetal growth impairment. However, treatment during gestational days 9–20 produced severe fetal hemorrhage, increased fetal resorption, and a significant reduction in litter size at day 21 of gestation. Approximately 36% of hemorrhagic lesions were externally visible in live fetuses, with many presenting as subcutaneous hemorrhages. More severe hemorrhages involved the brain, face, eyes, and occasionally the limbs. Intracranial hemorrhage, primarily intraventricular, was observed in 13% of exposed fetuses. Hemorrhage localized within the walls of the cerebral hemispheres resulted in focal areas of brain destruction, while hemorrhage affecting the eyes and ears caused tissue distortion and degeneration. Notably, classic skeletal features of human fetal warfarin syndrome, such as nasal hypoplasia and epiphyseal stippling, were not observed. The authors suggested that this difference may reflect species-specific developmental timing, as much of the skeletal ossification in rats occurs postnatally [61]. In a subsequent study, postnatal warfarin exposure in Sprague–Dawley rats was investigated by administering 100 mg/kg warfarin daily and simultaneously administering intramuscular injections of 10 mg/kg vitamin K1, beginning the day after birth and continuing for up to 12 weeks [62]. Treated rats demonstrated substantial growth impairment, including significant reductions in body weight, tail length, nasal length, and overall body length. Craniofacial abnormalities included shorter and broader snouts, smaller ear pinnae, and maxillonasal hypoplasia resulting from impaired skull growth. Significant reductions were observed in the dimensions of the skull, frontal bone, maxilla, premaxilla, nasal bone, and limb bones. Histological analysis revealed extensive calcification within the septal cartilage, particularly in the inferior half of the septum, with calcification extending throughout the full height of the anterior septum. These calcium deposits persisted for up to 15 months following treatment. Although classical stippling was absent, ectopic calcification formed “bridges” across growth plates, accompanied by disorganization and reduced cellularity within growth plate columns. These findings suggested that warfarin exposure disrupts normal cartilage maturation and endochondral ossification [62].

Additional evidence of warfarin-induced skeletal dysplasia was reported after administering daily warfarin doses of 0.05 mg/kg or 0.1 mg/kg to pregnant Wistar albino rats during gestational days 0–15, corresponding to the organogenesis period [63]. Fetuses exposed to warfarin exhibited incomplete ossification of the skull, enlarged fontanelles, incomplete development of the sacral and coccygeal vertebrae, abnormal metatarsal ossification, hind limb defects, and wavy ribs. At the higher dose, calcification was markedly impaired, and bone development was incomplete. Histopathological examination demonstrated hemorrhage and moderate degeneration within intervertebral tissues, while higher-dose exposure produced pronounced vertebral necrosis and degeneration [63]. Prenatal warfarin exposure in Sprague–Dawley rats treated daily from gestational days 8–22 was reported [64]. At 150 μg/kg, dams exhibited no external bleeding, and fetuses showed no gross abnormalities. However, higher doses of 185 and 200 μg/kg were lethal to the dams. At 175 μg/kg, maternal survival was reduced to approximately 57%, and fetuses demonstrated reductions in mandibular length, mandibular depth, and maxillary length, although these differences were not statistically significant after correction for fetal body weight. Histological analyses revealed widened hypertrophic zones in growth plates, disruption of the normal columnar arrangement of hypertrophic chondrocytes, and irregular cellular organization within cartilage. One dam exhibiting a markedly elevated prothrombin time produced fetuses with more severe skeletal abnormalities, including absence of ossification centers in the proximal and distal phalanges of both forelimbs and hind limbs, widened growth plates, and calcified hypertrophic zones. These findings further supported the relationship between excessive anticoagulation and impaired skeletal development [64]. The effects of therapeutic and toxic postnatal warfarin exposure in Sprague–Dawley rats were reported [65]. Rats received either a toxic dose consisting of 100 mg/kg warfarin combined with 10 mg/kg phylloquinone or a therapeutic dose of 0.07 mg/kg warfarin daily after birth. Neither treatment group demonstrated hemorrhage at the end of the study period. However, rats exposed to the toxic dose exhibited statistically significant reductions in skull and radius length. Animals receiving the therapeutic dose also demonstrated significantly reduced skull length, although radius length was not significantly affected (Table 11). These findings indicated that even therapeutic warfarin exposure may adversely affect craniofacial growth and skeletal development [65].

Rodent models offer improved translational relevance for studying warfarin embryotoxicity because they replicate key mammalian features, including maternal-fetal circulation, organogenesis, and skeletal development. Prenatal warfarin exposure in rats has reproduced several characteristics of fetal warfarin syndrome, such as fetal hemorrhage, intracranial bleeding, craniofacial abnormalities, growth impairment, and skeletal defects. Howe and Webster (1990) [61] reported that exposure during sensitive developmental windows resulted in severe fetal hemorrhage, including brain, facial, and ocular bleeding, along with increased fetal resorption. Further studies identified craniofacial hypoplasia, altered cartilage development, and impaired endochondral ossification, resembling key features of human warfarin embryopathy [62]. Rodent investigations have also advanced understanding of the developmental mechanisms underlying warfarin toxicity by linking disruption of vitamin K-dependent proteins with skeletal abnormalities. Warfarin exposure has been associated with reduced ossification of cranial structures, vertebral defects, delayed bone formation, and growth plate abnormalities, highlighting the importance of vitamin K pathways in skeletal development [63,64,65]. However, rodent models have limitations, as some hallmark human features, such as epiphyseal stippling, are inconsistently reproduced due to differences in skeletal maturation timing between rodents and humans. Therefore, rodent models provide important mechanistic and developmental insights but cannot fully capture the complete spectrum of human fetal warfarin syndrome.

### 5.4. Integrative Mechanisms Underlying Warfarin-Induced Developmental Toxicity Across Vertebrate Models

Across vertebrate models, evidence indicates that warfarin-induced developmental toxicity involves conserved biological pathways. By inhibiting vitamin K epoxide reductase (VKOR), warfarin decreases the availability of active vitamin K required for γ-carboxylation of vitamin K-dependent proteins. This disruption affects classical coagulation factors as well as non-coagulation proteins involved in bone formation, extracellular matrix organization, vascular integrity, and embryonic development. Impaired function of these proteins provides a mechanistic basis for the cartilage defects, reduced mineralization, skeletal abnormalities, and vascular disturbances observed in zebrafish and mammalian models [57,64]. In addition, zebrafish studies have revealed that developmental signaling pathways, particularly Wnt signaling involved in craniofacial patterning, contribute to warfarin-induced malformations and offer insights beyond observations possible from human clinical studies alone [55,57]. Collectively, animal models have enhanced understanding of fetal warfarin syndrome by enabling controlled assessment of exposure dose, developmental timing, affected tissues, and underlying molecular pathways. While human studies primarily characterize the clinical spectrum and establish associations between prenatal warfarin exposure and congenital abnormalities, experimental models allow investigation of causal mechanisms. The combined findings from zebrafish, chicken, and rodent models support the concept that fetal warfarin syndrome arises from disruption of conserved developmental processes involving vitamin K metabolism, skeletal morphogenesis, vascular development, and tissue patterning. These models provide an essential link between clinical manifestations and the molecular mechanisms responsible for warfarin-induced embryopathy.

Although current studies have established the importance of vitamin K-dependent mechanisms in warfarin-induced embryotoxicity, the effects of warfarin on broader developmental gene signaling networks remain incompletely understood. Further research is needed to determine how warfarin exposure influences known conserved pathways such as Sonic hedgehog (SHH), Bone Morphogenetic Protein (BMP), Wnt/β-catenin, Fibroblast Growth Factor (FGF), Notch, Transforming Growth Factor-β (TGF-β), and retinoic acid signaling pathways, etc., which regulate embryonic patterning, cell proliferation, differentiation, migration, and organogenesis in vertebrates. Investigating these pathways in vertebrate models will provide important insights into how disruption of vitamin K metabolism interacts with developmental gene networks to produce craniofacial, skeletal, cardiovascular, and other congenital abnormalities associated with fetal warfarin syndrome. Such studies may help identify key molecular targets and improve understanding of susceptibility mechanisms underlying warfarin-induced developmental toxicity.

## 6. Gene Signaling Networks in Warfarin-Induced Teratogenesis

Warfarin teratogenicity is primarily attributed to the disruption of vitamin K-dependent biochemical pathways required for normal embryonic development. Experimental studies in zebrafish, rodent, and avian models, as well as in cell culture systems, have identified alterations in signaling pathways regulating cartilage maturation, osteogenesis, vascular integrity, neuronal survival, and extracellular matrix organization following prenatal warfarin exposure. Importantly, several neurological manifestations associated with fetal warfarin syndrome have been reported prior to significant fetal coagulation factor expression, suggesting that mechanisms beyond anticoagulation-mediated hemorrhage contribute to developmental toxicity [66]. These observations support the hypothesis that warfarin may directly influence gene expression programs and signaling pathways critical for embryonic tissue differentiation and organogenesis. Understanding the molecular signaling networks affected by warfarin exposure is therefore essential for clarifying the pathogenesis of fetal warfarin syndrome and identifying potential targets for safer anticoagulation strategies during pregnancy.

### 6.1. γ-Glutamyl Carboxylase (GGCX), Thrombin, and Protease-Activated Receptor-1 (PAR-1) Regulate Vitamin K-Dependent Protein Activation

Warfarin inhibits vitamin K epoxide reductase complex subunit 1 (VKORC1), preventing the regeneration of the reduced form of vitamin K required for γ-glutamyl carboxylase (GGCX) activity. γ-Glutamyl carboxylase (GGCX) catalyzes the vitamin K-dependent γ-carboxylation of several proteins involved in coagulation and embryonic development. Known GGCX substrate proteins include coagulation-associated proteins such as prothrombin, protein C, protein S, protein Z, and coagulation factors VII, IX, and X, as well as developmental proteins including growth arrest-specific protein 6 (Gas6), matrix Gla protein (MGP), proline-rich γ-carboxylated proteins 1 and 2, nephrocalcin A–D, and the transmembrane proteins TMG3 and TMG4 [67]. Warfarin reduces vitamin K-mediated GGCX activity and subsequent γ-carboxylation of vitamin K-dependent proteins [68,69]. Experimental studies have demonstrated that impaired γ-carboxylation disrupts the activation of osteocalcin and matrix Gla protein (MGP), resulting in abnormal cartilage mineralization and skeletal dysplasia [70]. Mutations in *GGCX* produce phenotypes resembling fetal warfarin syndrome, including chondrodysplasia punctata and ectopic calcification [71]. While impaired γ-carboxylation is considered the central mechanism underlying fetal warfarin syndrome, increasing evidence suggests that warfarin exposure also disrupts broader developmental signaling networks involved in embryogenesis.

Warfarin-induced reduction in coagulation factors decreases thrombin generation and downstream PAR-1 signaling. PAR-1 regulates vascular stability, angiogenesis, and neural development during embryogenesis [72]. Experimental evidence suggests that impaired thrombin-PAR-1 signaling contributes to vascular fragility and fetal hemorrhage, particularly intracranial hemorrhage associated with CNS injury in fetal warfarin syndrome [73]. Although bleeding abnormalities are commonly observed in humans with γ-carboxylase mutations, developmental defects associated with non-coagulation γ-carboxylated proteins are comparatively rare. Experimental studies in mice have demonstrated the critical developmental importance of GGCX signaling. Homozygous *Ggcx*^-/-^ mice exhibit severe embryonic lethality, with approximately half of embryos dying between embryonic days 9.5 and 18.5, while surviving embryos succumb to intra-abdominal hemorrhage shortly after birth [67]. Interestingly, these embryos did not demonstrate ectopic calcification despite the absence of γ-carboxylation activity. Similar embryonic lethality has been observed in mice lacking protease-activated receptor-1 (*Par-1*), a thrombin receptor involved in vascular and developmental signaling. However, adult *Par-1*^-/-^ mice exhibit normal hemostasis, suggesting that mid-embryonic lethality in these models may result from impaired developmental signaling rather than hemorrhage alone. This finding supports the hypothesis that thrombin-mediated signaling pathways contribute to embryonic survival independently of their role in coagulation. In contrast, knockout models targeting several individual vitamin K-dependent proteins, including osteocalcin, Gas6, MGP, protein C, protein Z, and coagulation factors VII and IX, do not demonstrate embryonic lethality. Nevertheless, mutations in the *MGP* gene in humans cause Keutel syndrome, a disorder characterized by abnormal cartilage calcification, brachytelephalangia, and midfacial hypoplasia, phenotypes that overlap with the skeletal manifestations observed in fetal warfarin syndrome [67]. These findings suggest that disruption of multiple γ-carboxylated developmental pathways collectively contributes to the teratogenic effects of warfarin exposure.

### 6.2. Receptor Tyrosine Kinases (RTKs) and Eyk/Axl Signaling

Growth-arrest-specific protein 6 (Gas6) is a vitamin K-dependent protein (VKDP) that shares significant structural homology with protein S, a coagulation-associated protein affected by warfarin exposure. Gas6 functions as a ligand for the receptor tyrosine kinase (RTK) family members Tyro3, Axl, and MerTK, which regulate cellular proliferation, differentiation, survival, migration, and signal transduction during embryonic development [74,75]. Warfarin inhibits γ-carboxylation of GAS6, thereby reducing receptor activation and impairing signaling involved in endothelial survival, vascular stabilization, and neuronal development [76]. Experimental studies linked disrupted GAS6–AXL signaling to vascular instability and defective tissue remodeling. Because RTK signaling plays a central role in tissue morphogenesis and organogenesis, disruption of Gas6-mediated pathways has been proposed as a contributing mechanism in warfarin teratogenesis. Experimental studies using chicken embryo models demonstrated that warfarin exposure induces a dose-dependent reduction in tyrosine phosphorylation via pathways involving c-Eyk, the avian homolog of the Tyro3 receptor tyrosine kinase, which is widely expressed during embryogenesis [77]. Additional signaling molecules affected by warfarin exposure included pp125FAK (focal adhesion kinase), pp60c-src, and paxillin, all of which are critical regulators of cytoskeletal organization, signal transduction, and cellular adhesion during embryonic development. pp125FAK regulates cytoskeletal assembly and phosphorylates paxillin, a protein involved in actin-membrane interactions during organogenesis. pp60c-src functions in intracellular signaling pathways and is highly concentrated in developing neural tissue, whereas paxillin contributes to cell adhesion and morphogenesis [77]. Vitamin K administration increased phosphorylation of c-Eyk, pp125FAK, and paxillin and upregulated Src kinase activity in chicken embryos [78]. Warfarin exposure inhibited these vitamin K-mediated signaling responses, suggesting that disruption of RTK-associated phosphorylation pathways may contribute to impaired embryonic development during prenatal warfarin exposure. These findings support the hypothesis that warfarin teratogenicity extends beyond coagulation abnormalities to include disruption of developmental signaling networks that regulate cellular proliferation, cytoskeletal organization, and tissue morphogenesis.

### 6.3. Pregnane X Receptor (PXR) Pathway

Vitamin K functions as a specific ligand for the pregnane X receptor (PXR), a nuclear receptor that is a major regulator of xenobiotic metabolism and bile acid homeostasis [79]. In addition to its metabolic functions, PXR signaling plays important roles in skeletal development by regulating osteoblast differentiation and extracellular matrix (ECM)-associated genes, including *tsukushi* (*tsku*), *matrilin-2*, and *CD14* [59]. Altered PXR activity following warfarin exposure has been associated with impaired skeletal development and reduced ossification. Experimental studies have demonstrated that *PXR-*knockout mice develop osteopenia, underscoring the importance of PXR signaling in bone homeostasis and mineralization. In zebrafish models, warfarin exposure increased expression of the PXR-regulated genes *tsukushi* and *cyp3a65* [59]. The skeletal abnormalities observed in warfarin-exposed zebrafish have been proposed to result partly from tsukushi-mediated antagonism of bone morphogenetic protein (BMP) signaling, a pathway essential for skeletal patterning and osteogenesis. Disruption of BMP signaling may therefore contribute to impaired mineralization and skeletal dysplasia associated with prenatal warfarin exposure. The proposed interaction between Tsukushi and BMP signaling may also explain the observed in vivo upregulation of osteocalcin following warfarin exposure, which contrasts with findings reported in C2C12 cell culture models. These findings suggest that PXR-mediated signaling pathways may contribute to the skeletal manifestations of warfarin teratogenicity by modulating extracellular matrix organization and BMP-dependent developmental processes.

### 6.4. Sulfatide Metabolism and CNS Sequelae

Warfarin exposure has been shown to reduce the biosynthesis of sulfatides, essential sphingolipid components of myelin in the central nervous system [80]. Sulfatides play a critical role in myelin stability, neuronal signaling, and normal neural development. Disruption of sulfatide synthesis has therefore been proposed as a potential mechanism contributing to the neurological manifestations and developmental delays associated with fetal warfarin syndrome. Importantly, central nervous system abnormalities observed following prenatal warfarin exposure cannot be fully explained by hemorrhagic injury secondary to impaired coagulation. CNS abnormalities in a fetus exposed to warfarin between gestational weeks 8 and 12, a developmental period preceding significant fetal expression of coagulation factors [66]. These findings suggest that warfarin may directly interfere with neurodevelopmental pathways, independent of its anticoagulant effects, potentially by disrupting myelin-associated lipid metabolism and other developmental signaling mechanisms.

### 6.5. Ras Family Signaling Pathways

GTPases of the Ras protein family are critical regulators of embryonic development and participate in cellular processes including cell division, nuclear assembly, vesicle transport, cytoskeletal organization, and differentiation [81]. Ras signaling activity is tightly controlled by Ras-GTPase-activating proteins (Ras-GAPs), which accelerate the hydrolysis of active Ras-GTP to inactive Ras-GDP, thereby regulating downstream developmental signaling pathways [82]. One Ras-associated regulatory protein implicated in warfarin teratogenesis is SH3-domain binding protein (G3BP), which plays an essential role in embryogenesis and neurodevelopment. Experimental studies identified four redundant G3BP isoforms in embryonic stem cells, all of which were upregulated following warfarin exposure [81]. Warfarin-mediated disruption of GAS6/TAM receptor signaling alters downstream Ras-MAPK pathway activation, which is involved in cell proliferation, differentiation, and skeletal morphogenesis [83]. These findings suggest that warfarin may alter Ras-mediated signaling networks involved in cellular differentiation and neural development, potentially contributing to the neurodevelopmental abnormalities observed in fetal warfarin syndrome.

### 6.6. Wnt Signaling Pathway

Low-dose warfarin exposure has been shown to induce cleft palate formation in a subset of zebrafish embryos, producing phenotypes similar to the craniofacial abnormalities reported in human fetal warfarin syndrome. Experimental studies demonstrated that inhibition of Wnt signaling exacerbated the cleft palate phenotype, whereas treatment with Wnt agonists, including BIO, WAY-262611, and CHIR-99021, significantly rescued palatal development in affected embryos. Warfarin exposure was additionally associated with reduced expression of the canonical Wnt downstream transcription factors *tcf7* and *lef1*, indicating suppression of Wnt/β-catenin signaling activity [84]. This reduction was accompanied by decreased cellular proliferation and impaired viability of developing palatal structures. These findings suggest that disruption of canonical Wnt signaling contributes to warfarin-induced craniofacial malformations and may play an important role in the pathogenesis of cleft palate associated with prenatal warfarin exposure.

Transglutaminase 2 (TG2) has been identified as a critical mediator of warfarin-induced vascular calcification by activating canonical β-catenin signaling in vascular smooth muscle cells (VSMCs). Experimental studies in rat A10 VSMCs demonstrated that warfarin exposure promotes vascular calcification by activating the TG2/β-catenin signaling axis, thereby inducing osteogenic transformation of VSMCs. Inhibition of either TG2 activity or canonical β-catenin signaling significantly reduced warfarin-induced calcification, thereby identifying the TG2/β-catenin pathway as a potential therapeutic target for preventing warfarin-associated vascular calcification [85,86]. Disrupted Wnt signaling has been associated with delayed ossification, abnormal cartilage mineralization, and craniofacial defects characteristic of fetal warfarin syndrome.

### 6.7. Integrated Vitamin K-Dependent Mechanisms Underlying Warfarin Embryopathy

Warfarin induces teratogenic effects primarily through inhibition of vitamin K epoxide reductase complex subunit 1 (VKORC1), thereby preventing regeneration of vitamin K hydroquinone and limiting γ-glutamyl carboxylase (GGCX) activity required for post-translational modification of vitamin K-dependent proteins (VKDPs) [87,88]. This results in the accumulation of undercarboxylated VKDPs with reduced biological activity. Because VKDPs regulate morphological processes during embryo development, their coordinated dysfunction provides a mechanistic basis for the multisystem abnormalities observed in fetal warfarin syndrome [89,90].

Matrix Gla protein (MGP), a key non-coagulation VKDP, is a potent inhibitor of ectopic calcification and plays a central role in cartilage and bone development. Impaired γ-carboxylation of MGP disrupts endochondral ossification and extracellular matrix mineralization, contributing to chondrodysplasia punctata, nasal hypoplasia, and distal skeletal abnormalities characteristic of warfarin embryopathy [91,92]. Similarly, osteocalcin dysfunction affects osteoblast differentiation and bone matrix deposition, further compromising skeletal [93]. Phenotypic overlap between genetic defects in MGP or GGCX and warfarin exposure further supports this pathway-level mechanism [68,94].

Warfarin also interferes with γ-carboxylation of growth arrest-specific protein 6 (GAS6), reducing activation of the TAM receptor family (Tyro3, Axl, MerTK), which regulates cell survival, vascular stabilization, and neural development [95,96]. Disruption of GAS6–TAM signaling has been implicated in vascular fragility, impaired angiogenesis, and neurodevelopmental abnormalities [97,98]. In addition, vitamin K depletion influences broader developmental signaling networks, including PXR-mediated xenobiotic sensing, Wnt/β-catenin signaling in osteogenesis, Ras/MAPK pathways in cell proliferation, and thrombin–PAR-1 signaling in vascular and neural patterning [72,79,99]. Collectively, these interconnected pathways provide an integrated molecular framework linking VKOR inhibition to the skeletal, craniofacial, vascular, and neurological manifestations of fetal warfarin syndrome.

Despite these plausible connections relating warfarin with Vitamin K signaling, the direct molecular targets linking warfarin exposure to other known intracellular signaling networks regulating cell survival, proliferation, specification, differentiation and apoptosis remain largely unknown. It is unclear whether warfarin acts indirectly through metabolic stress, oxidative imbalance, or altered epigenetic regulation, or whether there are yet unidentified off-target interactions affecting developmental gene networks. Therefore, future research is essential to systematically map both upstream regulators and downstream transcriptional consequences of warfarin exposure beyond the vitamin K pathway. High-resolution multi-omics approaches, combined with functional studies in relevant embryonic models, will be critical to delineate how developmental pathways are integrated with xenobiotic response networks during warfarin-induced teratogenesis. This integrated framework will ultimately refine our understanding of fetal warfarin syndrome as a multi-pathway developmental disorder rather than a single-cascade vitamin K deficiency state.

## 7. Warfarin Derivatives and Alternative Anticoagulants

Several coumarin-derived anticoagulants with structural and pharmacological similarities to warfarin are used clinically, including phenprocoumon and acenocoumarol. These agents act by inhibiting vitamin K epoxide reductase, thereby impairing γ-carboxylation of vitamin K-dependent coagulation factors, via a mechanism similar to that of warfarin. Phenprocoumon is sometimes preferred in patients with poor warfarin metabolism because of its prolonged elimination half-life. The S- and R-enantiomers of phenprocoumon have half-lives of approximately 172 and 156 h, respectively, with the S-enantiomer accounting for most of the anticoagulant activity [16]. Structurally, phenprocoumon differs from warfarin by lacking the ketone group attached to the asymmetric carbon center [3]. Acenocoumarol, another coumarin derivative, has a substantially shorter half-life, with elimination half-lives of approximately 2 h for the S-enantiomer and 8 h for the R-enantiomer [16]. In contrast to warfarin, acenocoumarol contains a nitro group on the aromatic ring adjacent to the asymmetric carbon [3]. Despite pharmacokinetic differences, both phenprocoumon and acenocoumarol exert anticoagulant effects by inhibiting vitamin K-dependent pathways and may therefore share a teratogenic potential similar to that of warfarin. Indandione anticoagulants are another class of vitamin K antagonists with mechanisms of action comparable to those of warfarin. These compounds inhibit the synthesis of vitamin K-dependent clotting factors by interfering with vitamin K recycling pathways [100]. Although less commonly used today, indandione derivatives have historically been employed as oral anticoagulants and demonstrate similar anticoagulant and developmental toxicity profiles. Heparin and low-molecular-weight heparins (LMWHs) are widely used alternatives to vitamin K antagonists during pregnancy because they do not readily cross the placenta. Unlike warfarin (molecular weight 308.3 Da), which readily crosses the placenta, heparin is a large polysaccharide with an average molecular weight of 12–15 kDa and does not traverse the placental barrier, thereby minimizing fetal exposure. Comparatively, heparin exerts its anticoagulant effect by potentiating antithrombin III activity, thereby inhibiting thrombin and factor Xa [101,102]. Due to their limited placental transfer, heparin-based anticoagulants are generally considered safer for fetal development and are frequently recommended for anticoagulation management during pregnancy.

### Clinical Implications and Anticoagulation Strategies During Pregnancy

Management of anticoagulation during pregnancy remains a complex clinical challenge because treatment decisions must balance maternal protection from thromboembolic complications with potential fetal risks. Warfarin remains one of the most effective oral anticoagulants for preventing thromboembolic complications and valve thrombosis in patients with mechanical heart valves; however, because it crosses the placenta and interferes with vitamin K-dependent pathways, its use during pregnancy is associated with fetal risks, including fetal warfarin syndrome, particularly when exposure occurs during weeks 6–12 of gestation [103,104]. Current clinical guidelines recommend individualized anticoagulation strategies based on maternal thromboembolic risk, valve type, and patient preferences. The American College of Chest Physicians (ACCP), American College of Cardiology/American Heart Association (ACC/AHA), and European Society of Cardiology (ESC) guidelines recognize that warfarin provides superior maternal protection against mechanical valve thrombosis but requires careful consideration because of fetal [103,105,106]. In women requiring mechanical valve anticoagulation, continuation of warfarin at the lowest effective dose may be considered in selected high-risk patients, whereas alternative regimens are preferred when fetal risk reduction is prioritized. Low-molecular-weight heparin (LMWH) is frequently considered an alternative during pregnancy because it does not cross the placenta and therefore has minimal direct fetal exposure [103]. However, LMWH requires weight-adjusted dosing and monitoring of anti-factor Xa levels, and concerns remain regarding inadequate anticoagulation and increased risk of mechanical valve thrombosis, particularly in patients with older mechanical valves or additional thromboembolic risk factors [28,103]. Thus, anticoagulation decisions require careful evaluation of both maternal and fetal outcomes.

The clinical dilemma surrounding warfarin use reflects the competing risks of maternal thromboembolism and fetal developmental toxicity. While avoidance of warfarin during early pregnancy reduces the risk of embryopathy, interruption of effective anticoagulation may expose mothers to potentially life-threatening valve thrombosis or systemic embolism [103,104]. Therefore, individualized treatment plans involving cardiology, maternal–fetal medicine, and hematology specialists are recommended. Emerging strategies aim to improve anticoagulation safety during pregnancy through personalized dosing approaches, improved therapeutic monitoring, and development of anticoagulants with reduced placental transfer. However, direct oral anticoagulants (DOACs) are currently not recommended for patients with mechanical heart valves due to demonstrated risks of valve thrombosis and insufficient safety data during pregnancy [49,107,108,109,110,111]. Future research integrating clinical data with mechanistic insights from experimental models may improve prediction of fetal susceptibility and support safer anticoagulation approaches during pregnancy.

## 8. Conclusions

This review highlights the multifaceted nature of warfarin pharmacology, toxicity, and teratogenicity, emphasizing its continued clinical importance alongside its significant developmental risks during pregnancy. Although warfarin remains an effective and widely used anticoagulant, prenatal exposure can result in fetal warfarin syndrome (FWS), a complex disorder characterized by craniofacial, skeletal, neurological, cardiovascular, and growth abnormalities. Clinical and experimental evidence demonstrates that the severity of these phenotypes depends on the timing, duration, and dosage of exposure. Importantly, findings from zebrafish, chicken, and rodent models demonstrate that warfarin-induced developmental toxicity extends beyond hemorrhagic complications and involves disruption of multiple vitamin K-dependent molecular signaling pathways regulating cartilage formation, skeletal mineralization, vascular stability, cardiac morphogenesis, and neurodevelopment. Pathways involving GGCX, GAS6/TAM receptors, thrombin–PAR-1, PXR, Ras, and Wnt signaling appear to play critical roles in the pathogenesis of FWS. Collectively, these studies provide important mechanistic insight into the developmental effects of warfarin and support the need for careful anticoagulation management during pregnancy. Continued integration of developmental biology, pharmacogenomics, and experimental modeling will be essential for improving understanding of warfarin teratogenesis and for developing safer anticoagulant strategies with reduced fetal toxicity.

## Figures and Tables

**Table 1:** Physicochemical and pharmacological properties of Warfarin relevant to developmental exposure.

Property	Description/Value	Relevance to Developmental Toxicity
Chemical class	Synthetic 4-hydroxycoumarin derivative	Vitamin K antagonist responsible for anticoagulant and teratogenic effects [1,2,3].
Molecular formula	C_19_H_16_O_4_	Chemical identity [1].
Molecular weight	308.33 g/mol	Standard physicochemical characteristic [1].
Solubility	Poorly soluble in water; clinically formulated as the more water-soluble sodium salt	Influences oral absorption and systemic drug exposure [1].
pKa	5.0–5.2	Influences ionization, absorption, and placental transfer [1].
Plasma protein binding	>99% bound to albumin	Determines free circulating drug concentration and maternal pharmacokinetics [1].
Enantiomeric composition	Racemic mixture of R- and S-warfarin; S-warfarin is 3–5 times more potent.	Influences anticoagulant potency and pharmacokinetics [2].
Major metabolism	Hepatic metabolism primarily by CYP2C9 (S-warfarin); CYP1A2 and CYP3A4 (R-warfarin).	Maternal metabolism influences fetal drug exposure [2].
Placental transfer	Readily crosses the placenta	Directly exposes the embryo and fetus to warfarin during pregnancy [3].
Mechanism of action	Inhibits vitamin K epoxide reductase (VKOR), preventing regeneration of vitamin K hydroquinone.	Impairs γ-carboxylation of vitamin K-dependent proteins essential for coagulation and skeletal development, forming the basis of fetal warfarin syndrome [3].

**Abbreviations:** CYP, cytochrome P450; pKa, acid dissociation constant; VKOR, vitamin K epoxide reductase.

**Table 2:** Distribution of References Included in This Review by Publication Period.

Years	# of Refs	% of Total Refs
1970–1979	4	3.64
1980–1989	8	7.28
1990–1999	20	18.19
2000–2009	31	28.19
2010–2019	36	32.73
2020–2026	11	10
Total	110	100

**Table 4:** Skeletal and Musculoskeletal Abnormalities Reported Following Prenatal Warfarin Exposure in Humans.

Phenotype Category	Representative Clinical Findings	Representative Maternal Exposure	Typical Gestational Exposure	Evidence Strength	Representative References
Chondrodysplasia punctata (hallmark lesion)	Epiphyseal stippling involving vertebral, femoral, humeral, talar, tarsal, carpal, calcaneal, patellar, phalangeal, and pelvic epiphyses	3–12.5 mg/day	Primarily first-trimester exposure (weeks 6–12), although prolonged exposure has also been reported	Multiple case reports and case series	[27,29,30,32,34,35,36,38,39,41,42,43,47]
Digital abnormalities	Distal phalangeal hypoplasia	5–12.5 mg/day	Early gestation	Multiple case reports	[26,27,30,32,36,45]
Axial skeletal abnormalities	Cervical vertebral hypoplasia, kyphosis, short neck	5–11 mg/day	Throughout pregnancy or prolonged exposure	Case reports	[30,36,37,41]
Thoracic abnormalities	Pectus carinatum, pectus excavatum	Therapeutic doses	Variable	Case reports	[29,36,40]
Neuromuscular abnormalities	Depressed muscle tone	5–11 mg/day	Variable	Case reports	[29,30,33]

**Table 5:** Cardiorespiratory Phenotypes Associated with Prenatal Warfarin Exposure.

Phenotype Category	Representative Clinical Findings	Representative Maternal Exposure	Typical Gestational Exposure	Evidence Strength	Representative References
Congenital cardiac defects	ASD, VSD, PDA, aortic arch coarctation	5–6 mg/day (representative therapeutic range)	Most often reported following first-trimester exposure, with continued therapy in some cases	Predominantly case reports	[19,31,36,48,49]
Hematologic and circulatory findings	Anemia, preductal/postductal hypotension	5–6 mg/day	Variable	Case reports	[36,48]
Respiratory abnormalities	Respiratory distress	5–11 mg/day	Variable; reported after early or continued exposure	Multiple case reports	[29,30,38,41,43]

**Table 6:** Ophthalmologic Phenotypes Associated with Prenatal Warfarin Exposure.

Phenotype Category	Representative Clinical Findings	Representative Maternal Exposure	Typical Gestational Exposure	Evidence Strength	Representative References
Optic nerve abnormalities	Optic atrophy	~5 mg/day	Throughout pregnancy	Case reports	[26,29]
Anterior segment abnormalities	Corneal opacity, shallow anterior chamber, cataract	5–12.5 mg/day	Early or prolonged exposure	Case reports	[32,41]
Visual impairment	Blindness	Not consistently reported	Not consistently reported	Isolated case reports	[26,33]

**Table 7:** Neurodevelopmental and Central Nervous System Phenotypes Associated with Prenatal Warfarin Exposure.

Phenotype Category	Representative Clinical Findings	Representative Maternal Exposure	Typical Gestational Exposure	Evidence Strength	Representative References
Brain structural abnormalities	Hydrocephalus, ventriculomegaly, Dandy-Walker malformation, arachnoid/porencephalic cysts	Variable	Frequently associated with prolonged exposure beyond the first trimester	Multiple case reports	[26,31,35,42,47,48,50,51]
Hemorrhagic and ischemic lesions	Intracranial hemorrhage, subdural hematoma, basal ganglia calcification/ischemia	3.75–7.5 mg/day (reported cases)	Predominantly second and third trimester exposure	Case reports	[30,47,48,50,51,52]
Neurological dysfunction	Microcephaly, seizures, quadriplegia, language delay, intellectual disability, poor suck, reduced reflexes	Variable	Variable	Case reports and small case series	[29,30,32,33,37,46,51]

**Table 8:** Additional Developmental and Systemic Abnormalities Reported Following Prenatal Warfarin Exposure in Humans.

Phenotype Category	Representative Clinical Findings	Representative Maternal Exposure	Typical Gestational Exposure	Evidence Strength	Representative References
Pregnancy outcomes	Polyhydramnios, fetal growth restriction, low birth weight, fetal maceration	3–10 mg/day	Variable	Case reports and case series	[19,29,31,35,36,39,44,45,46]
Sensory abnormalities	Hearing loss, deafness	Not consistently reported	Not consistently reported	Case reports	[26,27,32,37]
Other developmental findings	Hypoplastic nails, delayed developmental milestones, prolonged prothrombin time	Variable	Variable	Case reports	[27,36,37,42]

**Table 9:** Developmental Abnormalities Observed in Zebrafish (*Danio rerio*) Following Warfarin Exposure.

Phenotype	Malformation Description	Reference
**Craniofacial**		
Craniofacial hypomineralization	Impaired mineralization of the dermal and facial cartilaginous structures, reducing hard tissue content in the cleithrum, basioccipital, parasphenoid, and ceratobrachial 5 bone.	Granadeiro (2019) [57].
Craniofacial cartilage reduction	Decreased cartilage content in growth plates of jaw and nasal bones with greatest impact in embryonic development.	Granadeiro (2019) [57].
Ceratohyal hypoplasia	Decreased length of throat-structural ceratohyal cartilage at 7 days post-fertilization.	Granadeiro (2019) [57].
**Circulation/Musculoskeletal**		
Delayed skeletal mineralization, generalized	Delayed skeletal ossification and mineral deposition during development.	Granadeiro (2019) [57].
Decreased vertebral mineralization	Reduced vertebral mineralization accompanied by decreased birefringence, indicative of impaired bone formation.	Granadeiro (2019) [57].
Tail malformation	Abnormal morphology of the tail.	Fernández (2014) [59].
Scoliosis	Sideways curvature of the spine observed as early as 1 day post-fertilization.	Strecker (2013) [56].
Cardiac edema	Accumulation of fluid within tissues secondary to impaired cardiac function.	Granadeiro (2019) [57].
Decrease in cardiac cone area	Impaired cross-sectional area of fusing myocardial precursors during development, which occurred around 20 h post-fertilization	Liu (2025) [58].
Cardiac hypoplasia	Underdevelopment of the heart characterized by reduced cardiac size and incomplete formation of cardiac structures, indicative of impaired cardiogenesis.	Fernández (2014) [59].
Pathological heart calcification	Calcified regions present adjacent to the aortic bulb	Fernández (2014) [59].
Cardiac looping defects	Defect in heart formation during the twisting of the cardiac tube to establish basic structure	Liu (2025) [58].
Impaired ventricle/atrium morphogenesis	Decreased size of the ventricle and atrium of the heart	Liu (2025) [58].
Absence of heartbeat (cardiac arrest)	Present at 3 days post-fertilization	Strecker (2013) [56].
Impaired circulation	Reduced or abnormal blood flow through the embryonic vasculature, indicative of disrupted cardiovascular function and compromised circulation during development.	Fernández (2014) [59].
**CNS/Neuro**		
Incomplete forebrain formation	Incomplete development of the forebrain observed following embryonic warfarin exposure, indicating disruption of normal central nervous system development.	Fernández (2014) [59].
Reduced ganglion cell/inner plexiform/inner nuclear layer ratios	Reduced retinal ganglion cell, inner plexiform layer, and inner nuclear layer thickness ratios, indicating impaired retinal development.	Granadeiro (2019) [57].
Brain hemorrhage	Intracranial hemorrhage observed predominantly in the highest exposure group.	Fernández (2014) [59].
**Other**		
Growth retardation	Reduced body size and growth compared with controls at 16 days post-fertilization following embryonic exposure, though endotrophic exposure caused growth reduction in all dose groups.	Granadeiro (2019) [57], Strecker (2013) [56].
Hemorrhage, generalized	Bleeding throughout the body, observed in the highest-dosage group.	Granadeiro (2019) [57].
Swim bladder reduction	Reduction in size of the gas-filled swim bladder in the highest-dosage group.	Granadeiro (2019) [57].
Hepatocellular shrinkage	Reduction in size of liver mesenchymal cells.	Granadeiro (2019) [57].
Yolk deformity	Altered composition and/or size of yolk, presence of edema in yolk.	Strecker (2013) [56].
Underdeveloped somites	Underdevelopment of mesoderm blocks which differentiate into skeletal and connective tissue.	Fernández (2014) [59].

**Table 10:** Phenotypic Effects of Postnatal Warfarin Exposure in Chickens.

Phenotype Category/Effect	Description	Reference
**Circulation/Musculoskeletal:** Intramuscular hemorrhage	Dose-dependent hemorrhage is observed in the major and minor pectoralis, biceps femoris, and sartorius muscles, as well as the intraperitoneal region.	Veltmann (1981) [60].
**Growth:** Decreased body weight	Reduced feed consumption resulting in decreased body weight gain.	Veltmann (1981) [60].
Hematological: Prolonged prothrombin time	Increased prothrombin time, with peak effects generally observed one week after exposure; in broiler chicks receiving 100 ppm warfarin, peak prolongation occurred after three weeks.	Veltmann (1981) [60].

**Table 11:** Developmental and Skeletal Abnormalities Associated with Warfarin Exposure in Rats.

Phenotype	Malformation Description	Reference
**Craniofacial**		
Maxillonasal Hypoplasia	Nasal bone length was reduced by 11–13% compared with controls.	Howe (1992) [62]
Reduced skull length	Cranial shortening, with the anterior skull region more severely affected; total skull length reduced by 15.7 ± 2.3% relative to controls.	Howe (1992) [62], Chetot (2020) [65]
Shortened and broadened snout	Altered snout morphology characterized by reduced length and increased width.	Howe (1992) [62]
Reduced pinna size	Decreased size of the external ear (pinna).	Howe (1992) [62]
Decreased mandibular length	Reduction in mandibular length; differences were not significant after adjustment for fetal body weight.	Feteih (1990) [64]
Decreased maxillary length	Reduced maxillary and premaxillary dimensions; maxilla and premaxilla decreased by approximately 6–12% and 5–7%, respectively.	Feteih (1990) [64], Howe (1992) [62]
Septal cartilage calcification	Premature or abnormal calcification of the nasal septal cartilage.	Howe (1992) [62]
**Craniofacial/Skeletal**		
Incomplete ossification of the skull	Delayed or incomplete cranial bone ossification.	Abdulsamad (2023) [63]
Enlarged fontanelles	Large persistent fontanelles resulting from delayed cranial ossification.	Abdulsamad (2023) [63]
**Circulatory/Hemorrhagic**		
Subcutaneous hemorrhage	Hemorrhage within subcutaneous tissues; represented a substantial proportion of observed bleeding events.	Howe (1990) [61]
Brain hemorrhage	Predominantly intraventricular hemorrhage, observed in approximately 13% of fetuses.	Howe (1990) [61]
Facial hemorrhage	Hemorrhage affecting facial soft tissues.	Howe (1990) [61]
Ocular hemorrhage	Hemorrhage involving periocular or ocular tissues.	Howe (1990) [61]
Limb hemorrhage	Hemorrhage affecting limb soft tissues.	Howe (1990) [61]
Musculoskeletal/Skeletal		
Reduced radius length	Radius length was 11.3 ± 0.6% reduced. Postnatal exposure.	Chetot (2020) [65]
Reduced tail length	12–17% reduction. Postnatal exposure.	Howe (1992) [62]
Reduced forelimb bone length	Forelimb bones shortened by approximately 4–5%.	Howe (1992) [62]
Wavy ribs	Abnormal rib curvature and morphology.	Abdulsamad (2023) [63]
Generalized incomplete bone development	Extensive reduction in skeletal mineralization and calcification.	Abdulsamad (2023) [63]
Vertebral necrosis and degeneration	Marked vertebral degeneration and necrosis observed in the high-dose group.	Abdulsamad (2023) [63]
Growth/Development		
Growth restriction	Postnatal exposure. Generalized reduction in fetal growth and development.	Howe (1992) [62]
Reduced body weight	Body weight is reduced by approximately 7–13% compared with controls.	Howe (1992) [62]
Reduction in cell count or disorganization of growth plate column cells	Reduced numbers and disorganization of growth plate columnar chondrocytes.	Howe (1992) [62]
Disorganized hypertrophic zones	Hypertrophic cartilage zones were widened, calcified, and disorganized, occupying a greater proportion of total cartilage length than in controls. Mean width was 18.3% of total cartilage length compared to control (14.2%).	Feteih (1990) [64]

## Data Availability

No new data were created or analyzed in this study. Data sharing is not applicable to this article.

## Supplementary Materials

The following supporting information can be downloaded at: https://www.mdpi.com/article/10.3390/jdb14030034/s1, Table S1: Pharmacological properties of selected warfarin analogues reported in experimental studies; Table S2: Comprehensive Summary of Craniofacial Phenotypes Reported Following Prenatal Warfarin Exposure in Humans; Table S3: Comprehensive Summary of Skeletal and Musculoskeletal Abnormalities Reported Following Prenatal Warfarin Exposure in Humans; Table S4: Comprehensive Summary of Cardiovascular and Respiratory Phenotypes Reported Following Prenatal Warfarin Exposure in Humans; Table S5: Comprehensive Summary of Ophthalmologic Phenotypes Reported Following Prenatal Warfarin Exposure in Humans; Table S6: Comprehensive Summary of Neurodevelopmental and Central Nervous System Phenotypes Reported Following Prenatal Warfarin Exposure in Humans; Table S7: Comprehensive Summary of Additional Developmental and Systemic Abnormalities Reported Following Prenatal Warfarin Exposure in Humans.
