# Supplementary material for: Warfarin-Induced Developmental Toxicity: Insights into Embryogenesis, Teratogenicity, and Molecular Pathways
Source: J Dev Biol. 2026 Aug 1;14(3):34. doi: 10.3390/jdb14030034 (PMC13511227; doi:10.3390/jdb14030034)
Supplement: Supplementary file 1 [file jdb-14-00034-s001.zip › jdb-4396942-supplementary.pdf]

Supplementary Tables:

Table S1. Pharmacological properties of selected warfarin analogues reported in experimental studies.

<table>
<tr><th>Name</th><th>Mol. Weight (g/mol)</th><th colspan="2">Pharmacological Observations</th><th>Source</th></tr>
<tr><td>Warfarin</td><td>308.3</td><td colspan="2">Parent compound used as comparator</td><td>PubChem [1]</td></tr>
<tr><td>6-hydroxywarfarin</td><td>324.3</td><td colspan="2" rowspan="3">Significantly earlier minimum mean percent of normal prothrombin complex activity (%PCA), with activity prior to mean similar to regular warfarin (in rabbit model).</td><td rowspan="6">Obaseki (1987) [109]</td></tr>
<tr><td>7-hydroxywarfarin</td><td>324.3</td></tr>
<tr><td>8-hydroxywarfarin</td><td>324.3</td></tr>
<tr><td>4′-hydroxywarfarin</td><td>324.3</td><td colspan="2">Similar to other hydroxy-analogs with the exception of half the minimum mean %PCA (in rabbit model).</td></tr>
<tr><td>6-chlorowarfarin</td><td>342.77</td><td colspan="2" rowspan="2">Decrease in ‘real’ anticoagulant activity, minimum mean %PCA was higher and occurred around 2/3rds of a day sooner, although activity to that point mirrored that of regular warfarin (in rabbit model).</td></tr>
<tr><td>6-bromowarfarin</td><td>387.22</td></tr>
<tr><td>6-fluorowarfarin</td><td>326.32</td><td>IC_{50} was 0.79 times R-warfarin in rat microsomes. Tighter HSA binding than S-warfarin. Increased PT time comparable or longer than S-warfarin.</td><td rowspan="3">Not metabolized to 6- or 7-hydroxywarfarin by CYP2C9, despite binding with similar high affinities (measured in human liver microsomes with competitive inhibition of S-warfarin binding).</td><td rowspan="2">Zhang (1997) [111], Kerr (1997) [108]</td></tr>
<tr><td>7-fluorowarfarin</td><td>326.32</td><td>IC_{50} was 0.87 times R-warfarin in rat microsomes.</td></tr>
<tr><td>6,7,8-trifluorowarfarin</td><td>362.30</td><td></td><td>Zhang (1997) [111]</td></tr>
<tr><td>^11C alcohol analog</td><td>324.37</td><td colspan="2">IC_{50} was twice that of R-warfarin in rat microsomes. Did not increase PT time above controls at 1600 ug/day.</td><td rowspan="3">Kerr (1997) [108]</td></tr>
<tr><td>4C $COCH_3$ ether substituent</td><td>350.4</td><td colspan="2">Increased prothrombin time (PT) time comparable or longer than S-warfarin. IC_{50} was 1.70 times that of R-warfarin.</td></tr>
<tr><td>4C $CO(CH_2)_2CH_3$ ether substituent</td><td>378.4</td><td colspan="2">IC_{50} was 1.80 times that of R-warfarin.</td></tr>
</table>

Note: *Molecular weights were calculated from the molecular formulas of the respective compounds using standard atomic masses.*

Table S2. Comprehensive Summary of Craniofacial Phenotypes Reported Following Prenatal Warfarin Exposure in Humans

Phenotype	Key Findings	Sources	Representative Exposure Range	Timeline
Hypoplasia				
Nasal hypoplasia:	Incomplete development of these regions, leading to affected morphology	Stevenson (1980) [29], Gupta (2010) [43], Howe (1997) [30], Harrod (1981) [32], Birbal (2023) [34], Tongsong (1999) [35], Hou (2004) [36], Ferreira (2018) [38], Dilli (2011) [41], Vitale (1999) [19], Pauli (1987) [27], Basu (2016) [42], Vilhena (2015) [40], Chan (2003) [31], Raghav (2007) [37].	5 mg/day [29, 43, 34, 36, 38]<br>5 mg/day alternating with 10 mg/day [41]<br>11 mg/day, reduced to 6 mg/day [30]<br>12.5 mg/day [32]<br>10 mg/day [35].	Throughout pregnancy [29, 36, 41, 35], Until 9 gestational weeks then from 12 gestational weeks onwards [43], Until 4 gestational weeks then from 12 gestational weeks onward [34], Until 6 gestational weeks then from 12 gestational weeks onward [38],<br>Reduced dose mid gestation discontinued at 2 weeks before delivery [30], From the 6th through 24th week of gestation [32].
Maxillary hypoplasia:		Howe (1997) [30]	11 mg/day, reduced to 6 mg/day [30].	Reduced dose mid gestation discontinued at 2 weeks before delivery [30].
Midfacial hypoplasia:		Barr (1976) [45], Pati (1994) [46].	7.5 mg/day [45].	Discontinued one week prior [illegible] delivery [45].
Mandibular hypoplasia:		Chan (2003) [31], Stevenson (1980) [29].	5 mg/day [29].	Throughout pregnancy [29].
Microstomia	Reduced size of the oral opening, causing difficulty when opening the mouth	Chan (2003) [31].		
Absent nasal bones	Lack of nasal bones in the facial structure	Ferreira (2018) [38], Mehndiratta (2010) [39], Basu (2016) [42].	5 mg/day [38]<br>3 mg/day [39].	Until 6 gestational weeks, then from 12 gestational weeks onward [38], throughout pregnancy [39].
Saddle nose	Collapse or deformity of the nasal bridge, creating a saddle-like appearance	Stevenson (1980) [29], Chan (2003) [31], Harrod (1981) [32], Tongsong (1999) [35], Hou (2004) [36], Mehndiratta (2010) [39], Vitale (1999) [19], Basu (2016) [42], Dilli (2011) [41], Songmen (2017) [44], Vilhena (2015) [40], Wainwright (2010) [47].	5 mg/day [29]<br>5-6 mg/day [44]<br>10 mg/day [35]<br>3 mg/day [39]<br>5 mg/day alternating with 10 mg/day [41].	Throughout pregnancy [29, 35, 36, 39, 41], Until 35 weeks of gestation [44].
Short or small nose	Reduced length or profile of the nose	Vilhena (2015) [40], Wainwright (2010) [47], Howe (1997) [30], Hou (2004) [36], Pauli (1987) [27], Dilli (2011) [41].	11 mg/day, reduced to 6 mg/day [30]<br>5 mg/day [36].	Reduced dose mid gestation discontinued at 2 weeks before delivery [30], Throughout pregnancy [36].
Nasal stenosis	Weakening or narrowing of	Stevenson (1980) [29],	5 mg/day [29, 43]	Throughout pregnancy [29],

	the nasal passages	Gupta (2010) [43], Chan (2003) [31], Songmen (2017) [44].	5-6 mg/day [44].	Until 35 weeks of gestation [44], Until 9 gestational weeks then from 12 gestational weeks onwards [43].
Deep ala nasi groove	Pronounced groove between the ala and the tip of the nose	Stevenson (1980) [29], Hou (2004) [36], Pauli (1987) [27], Basu (2016) [42], Dilli (2011) [41], Songmen (2017) [44].	5 mg/day [29, 36] 5-6 mg/day [44].	Throughout pregnancy [29, [36, 41], Until 35 weeks of gestation [44].
Anteverted nostrils	Front- and/or upward-facing nostrils	Stevenson (1980) [29], Basu (2016) [42].	5 mg/day [29].	Throughout pregnancy [29].
Laryngomalacia with tracheomalacia	Weakness and lacking rigidity of the tracheal cartilage and the tissue covering the vocal cords	Hou (2004) [36], Basu (2016) [42].	5 mg/day [36].	Throughout pregnancy [36].
Choanal atresia	Blockage of the posterior nasal passage by soft tissue or bone formation	Chan (2003) [31], Songmen (2017) [44].	5-6 mg/day [44].	Until 35 weeks of gestation [44].
Flattening of the face	Depression of facial structures creating a diminished side profile	Chan (2003) [31], Tongsong (1999) [35].	10 mg/day [35].	Throughout pregnancy [35].
Hypertelorism	Increased intercanthal (between the eyes) distance	Barr (1976) [45], Dilli (2011) [41], Songmen (2017) [44].	7.5 mg/day [45] 5 mg/day alternating with 10 mg/day [41] 5-6 mg/day [44].	Throughout pregnancy [41] Until 35 weeks of gestation [44] Discontinued one week prior to delivery [45].
Auricular fold hypoplasia	'Cupped ear' presentation where folds inside the pinna have failed to fully develop	Chan (2003) [31].		
Pinnae hypoplasia	Incomplete development of the external ear cartilage	Chan (2003) [31].		
Flattening of the occipital area		Chan (2003) [31].		
Macrocephaly		Tongsong (1999) [35], Stevenson (1980) [29].	10 mg/day [35] 5 mg/day [29].	Throughout pregnancy [35, 29].

Note: *Gestational exposure periods are reported as described in the original studies. Craniofacial abnormalities are most commonly associated with warfarin exposure during weeks 6–12 of gestation, although abnormalities have also been reported following exposure throughout pregnancy.*

Table S3. Comprehensive Summary of Skeletal and Musculoskeletal Abnormalities Reported Following Prenatal Warfarin Exposure in Humans

**Phenotype**	**Key Findings**	**Sources**	**Representative Exposure Range**	**Timeline**
Pectus carinatum deformity	Anteriorly protruding sternum and ribs	Stevenson (1980) [29], Hou (2004) [36].	5 mg/day [29, 36].	Throughout pregnancy [29, 36].
Pectus excavatum	Posterior displacement of the sternum leading to a sunken-chest appearance	Vilhena (2015) [40].		
Depressed muscle tone	Low passive muscle resistance	Stevenson (1980) [29], [D] Howe (1997) [30],	5 mg/day [29] 11 mg/day, reduced	Throughout pregnancy [29], Reduced dose mid gestation

		Sherman (1976) [33].	to 6 mg/day [30].	discontinued at 2 weeks before delivery [30].
Chondrodysplasia punctata (epiphyseal stippling)				
Unspecified	Appearance of 'dots' of calcification in the cartilaginous growth plates of bones and other cartilage tissue	Gupta (2010) [43], Birbal (2023) [34], Wainwright (2010) [47].	5 mg/day [43, 34].	Until 4 gestational weeks then from 12 gestational weeks onward [34], Until 9 gestational weeks then from 12 gestational weeks onwards [43].
Vertebral epiphyses		Stevenson (1980) [29], Howe (1997) [30], Harrod (1981) [32], Tongsong (1999) [35], Hou (2004) [36], Pauli (1987) [27], Basu (2016) [42], Dilli (2011) [41].	5 mg/day [29]<br>5 mg/day alternating with 10 mg/day [41]<br>11 mg/day, reduced to 6 mg/day [30]<br>12.5 mg/day [32].	Throughout pregnancy [29, 41, 35, 36], Reduced dose mid gestation discontinued at 2 weeks before delivery [30], From the 6th through 24th week of gestation [32].
Talus / ankle epiphyses		Howe (1997) [30], Basu (2016) [42], Dilli (2011) [41].	11 mg/day, reduced to 6 mg/day [30]<br>5 mg/day alternating with 10 mg/day [41].	Reduced dose mid gestation discontinued at 2 weeks before delivery [30], Throughout pregnancy [41].
Humerus		Howe (1997) [30], Basu (2016) [42].	11 mg/day, reduced to 6 mg/day [30].	Reduced dose mid gestation discontinued at 2 weeks before delivery [30].
Carpal bones		Howe (1997) [30].	11 mg/day, reduced to 6 mg/day [30].	Reduced dose mid gestation discontinued at 2 weeks before delivery [30].
Elbow epiphyses		Howe (1997) [30].	11 mg/day, reduced to 6 mg/day [30].	Reduced dose mid gestation discontinued at 2 weeks before delivery [30].
Distal tibia		Howe (1997) [30].	11 mg/day, reduced to 6 mg/day [30].	Reduced dose mid gestation discontinued at 2 weeks before delivery [30].
Femur epiphyses		Chan (2003) [31], Tongsong (1999) [35], Ferreira (2018) [38], Mehndiratta (2010) [39], Basu (2016) [42].	10 mg/day [35]<br>5 mg/day [38]<br>3 mg/day [39].	Throughout pregnancy [35, 39], Until 6 gestational weeks then from 12 gestational weeks onward [38].
Patellar epiphyses		Basu (2016) [42].		
Hips and Pelvic bones		Basu (2016) [42], Chan (2003) [31], Howe (1997) [30].	11 mg/day, reduced to 6 mg/day [30].	Reduced dose mid gestation discontinued at 2 weeks before delivery [30].
Tarsal epiphyses		Chan (2003) [31], Harrod (1981) [32], Basu (2016) [42].	12.5 mg/day [32].	From the 6th through 24th week of gestation [32].
Calcaneal epiphyses		Harrod (1981) [32], Hou (2004) [36], Mehndiratta (2010) [39].	12.5 mg/day [32]<br>5 mg/day [36]<br>3 mg/day [39].	From the 6th through 24th week of gestation [32], Throughout pregnancy [36, 39].

Phalangeal epiphyses		Harrod (1981) [32], Raghav (2007) [37], Basu (2016) [42].	12.5 mg/day [32].	From the 6th through 24th week of gestation [32].
Epiphyseal and paraspinal punctate calcification	Punctate calcification within cartilage and adjacent tissue	Birbal (2023) [34].	5 mg/day [34].	Until 4 gestational weeks then from 12 gestational weeks onward [34].
Distal phalangeal hypoplasia	Underdevelopment in the distal finger and toe regions	Howe (1997) [30], Harrod (1981) [32], Hou (2004) [36], Pauli (1987) [27], Hall (1980) [26], Barr (1976) [45].	12.5 mg/day [32]<br>11 mg/day, reduced to 6 mg/day [30]<br>7.5 mg/day [45]<br>5 mg/day [36].	From the 6th through 24th week of gestation [32], Reduced dose mid gestation discontinued at 2 weeks before delivery [30], Discontinued one week prior to delivery [45], Throughout pregnancy [36].
Cervical vertebra hypoplasia	Underdevelopment of the cervical vertebrae	Howe (1997) [30].	11 mg/day, reduced to 6 mg/day [30].	Reduced dose mid gestation discontinued at 2 weeks before delivery [30].
Kyphosis	Hunch-back posture caused by excess outwards rounding of the spine	Howe (1997) [30], Raghav (2007) [37].	11 mg/day, reduced to 6 mg/day [30].	Reduced dose mid gestation discontinued at 2 weeks before delivery [30].
Short neck		Hou (2004) [36], Dilli (2011) [41].	5 mg/day [36]<br>5 mg/day alternating with 10 mg/day [41].	Throughout pregnancy [36, 41].

Note: *Epiphyseal stippling (chondrodysplasia punctata) represents the most characteristic skeletal manifestation of fetal warfarin syndrome and may involve multiple skeletal regions. Dosages and gestational exposure periods are reported as described in the original studies.*

Table S4. Comprehensive Summary of Cardiovascular and Respiratory Phenotypes Reported Following Prenatal Warfarin Exposure in Humans

Phenotype	Key Findings	Sources	Representative Exposure Range	Timeline
Atrial Septal Defect (ASD)	Abnormal opening in the interatrial septum permitting left-to-right shunting of blood between the atria.	Hou (2004) [36], Shil (2020) [110].	5 mg/day [36]<br>6 mg/day followed by 5 mg/day [90].	Throughout pregnancy [36], Until 10 weeks of gestation then from 14 weeks onward [90].
Ventricular Septal Defect (VSD)	Abnormal opening in the interventricular septum permitting left-to-right shunting of blood between the ventricles.	Chan (2003) [31], Starling (2012) [50], Vitale (1999) [19], Shil (2020) [110].	5-6 mg/day [50]<br>6 mg/day followed by 5 mg/day [90].	Until 17 weeks then from 23 weeks onward [50], Until 10 weeks of gestation then from 14 weeks onward [90].
Patent Ductus Arteriosus (PDA)	Persistence of the fetal ductus arteriosus after birth, resulting in abnormal blood flow between the aorta and pulmonary artery.	Hou (2004) [36].	5 mg/day [36].	Throughout pregnancy [36].
Aortic Arch Coarctation	Congenital narrowing of a segment of the aorta, typically involving the aortic arch or descending aorta.	Chan (2003) [31], Starling (2012) [50].	5-6 mg/day [50].	Until 17 weeks then from 23 weeks onward [50].

Preductal / postductal hypotension	Differential blood pressure and/or oxygen saturation between preductal and postductal circulations, suggestive of congenital cardiovascular pathology.	Starling (2012) [50].	5-6 mg/day [50].	Until 17 weeks then from 23 weeks onward [50].
Anemia	Reduced hemoglobin concentration or red blood cell mass.	Starling (2012) [50], Hou (2004) [36].	5-6 mg/day [50] 5 mg/day [36].	Until 17 weeks then from 23 weeks onward [50], Throughout pregnancy [36].
Respiratory distress	Respiratory compromise characterized by labored breathing, tachypnea, or inadequate oxygenation.	Stevenson (1980) [29], Gupta (2010) [43], Howe (1997) [30], Ferreira (2018) [38], Dilli (2011) [41].	5 mg/day [29, 43, 38] 5 mg/day alternating with 10 mg/day [41] 11 mg/day, reduced to 6 mg/day [30].	Throughout pregnancy [29, 41], Until 9 gestational weeks then from 12 gestational weeks onwards [43], Until 6 gestational weeks then from 12 gestational weeks onward [38], Reduced dose mid gestation discontinued at 2 weeks before delivery [30].

Table S5. Comprehensive Summary of Ophthalmologic Phenotypes Reported Following Prenatal Warfarin Exposure in Humans

Phenotype	Key Findings	Sources	Representative Exposure Range	Timeline
Optic atrophy	Degeneration of the optic nerve resulting in visual impairment or vision loss.	Stevenson (1980) [29], Hall (1980) [26].	5 mg/day [29].	Throughout pregnancy [29].
Corneal opacity	Loss of corneal transparency that may impair the transmission of light to the retina.	Harrod (1981) [32].	12.5 mg/day [32].	From the 6th through 24th week of gestation [32].
Shallow anterior chamber	Reduced depth of the anterior chamber between the cornea and iris.	Dilli (2011) [41].	5 mg/day alternating with 10 mg/day [41].	Throughout pregnancy [41].
Cataract	Opacification of the crystalline lens resulting in impaired vision.	Harrod (1981) [32].	12.5 mg/day [32].	From the 6th through 24th week of gestation [32].
Blindness	Partial or complete loss of vision.	Sherman (1976) [33], Hall (1980) [26].	Not reported	Not reported

Table S6. Comprehensive Summary of Neurodevelopmental and Central Nervous System Phenotypes Reported Following Prenatal Warfarin Exposure in Humans

Phenotype	Key Findings	Sources	Representative Exposure Range	Timeline
Microcephaly	Abnormally reduced head circumference resulting from impaired brain growth.	Sherman (1976) [33], Pati (1994) [46].	Not reported	Not reported

Sluggish pupil / poor pupillary response	Delayed or diminished pupillary constriction in response to light.	Harrod (1981) [32].	12.5 mg/day [32].	From the 6th through 24th week of gestation [32].
Poor suck	Impaired neonatal sucking reflex affecting feeding ability.	Stevenson (1980) [29], Matar (2016) [49].	5 mg/day [29].	Throughout pregnancy [29].
Absent or decreased deep-tendon reflexes	Reduced or absent neuromuscular reflex responses elicited by tendon percussion.	Stevenson (1980) [29], Howe (1997) [30].	5 mg/day [29]<br>11 mg/day, reduced to 6 mg/day [30].	Throughout pregnancy [29], Reduced dose mid gestation discontinued at 2 weeks before delivery [30].
Spinal cord thinning	Reduced spinal cord diameter or volume.	Raghav (2007) [37].	Not reported	Not reported
Spinal cord compression	Application of pressure to the spinal cord	Raghav (2007) [37].	Not reported	Not reported
Spina bifida	Congenital neural tube defect resulting from incomplete closure of the vertebral arches.	Vitale (1999) [19].	Not reported	Not reported
Ventriculomegaly	Abnormal enlargement of the cerebral ventricles.	Tongsong (1999) [35], Lee (2003) [48], Pati (1994) [46].	10 mg/day [35]<br>7.5 mg/day, then 7 mg/day warfarin [48].	Throughout pregnancy [35], Until 5 weeks of gestation then from 14 weeks onward [48].
Frontal cystic lesions	Cystic lesions involving the frontal cerebral region.	Starling (2012) [50].	5-6 mg/day [50].	Until 17 weeks then from 23 weeks onward [50].
Arachnoid cyst	Fluid-filled sac located between the arachnoid mater and central nervous tissue	Lee (2003) [48].	7.5 mg/day, then 7 mg/day warfarin [48].	Until 5 weeks of gestation then from 14 weeks onward [48].
Dandy-Walker malformation	Congenital malformation involving hypoplasia of the cerebellar vermis and cystic dilation of the fourth ventricle.	Chan (2003) [31], Hall (1980) [26], Basu (2016) [42].	Not reported	Not reported
Porencephalic cyst	Cystic cavity within the cerebral hemisphere, typically resulting from focal brain injury.	Matar (2016) [49].	Not reported	Not reported
Hemorrhagic posterior fossa cyst	Hemorrhagic cystic lesion located within the posterior cranial fossa.	Matar (2016) [49].	Not reported	Not reported
Spinal cord lesion	Spinal cord tissue alteration or damage	Howe (1997) [30].	11 mg/day, reduced to 6 mg/day [30].	Reduced dose mid gestation discontinued at 2 weeks before delivery [30].
Intracranial hemorrhage	Bleeding within the cranial cavity.	Lee (2003) [48], Matar (2016) [49], Shan (2023) [51], Wainwright (2010) [47].	7.5 mg/day, then 7 mg/day warfarin [48]<br>3.75 mg/day [51].	Until 5 weeks of gestation then from 14 weeks onward [48], Until 5 gestational weeks then from 16 gestational weeks onward [51].
Intracranial mass	Space-occupying intracranial lesion identified by neuroimaging.	Lee (2003) [48].	7.5 mg/day, then 7 mg/day warfarin [48].	Until 5 weeks of gestation then from 14 weeks onward [48].
Ischemic	Ischemic injury involving the	Howe (1997) [30].	11 mg/day, reduced	Reduced dose mid gestation

changes in basal ganglia	basal ganglia.		to 6 mg/day [30].	discontinued at 2 weeks before delivery [30].
Subdural hematoma	Accumulation of blood between the brain and the dura mater	Lee (2003) [48], Matar (2016) [49].	7.5 mg/day, then 7 mg/day warfarin [48].	Until 5 weeks of gestation then from 14 weeks onward [48].
Dystrophic calcification associated with white matter necrosis	Pathological calcium deposition associated with cerebral white matter necrosis.	Wainwright (2010) [47].	Not reported	Not reported
Calcified basal ganglia	Abnormal calcium deposition within the basal ganglia.	Starling (2012) [50].	5-6 mg/day [50]	Until 17 weeks then from 23 weeks onward [50].
Quadriplegia	Paralysis affecting all four limbs and the trunk.	Howe (1997) [30].	11 mg/day, reduced to 6 mg/day [30]	Reduced dose mid gestation discontinued at 2 weeks before delivery [30].
Hydrocephalus	Abnormal accumulation of cerebrospinal fluid within the cerebral ventricular system.	Chan (2003) [31], Matar (2016) [49], Wainwright (2010) [47].	Not reported	Not reported
Language Delay	Delayed acquisition of age-appropriate language abilities.	Harrod (1981) [32].	Not reported	Not reported
Intellectual disability, unspecified	Impairment of intellectual functioning and adaptive behavior.	Sherman (1976) [33], Raghav (2007) [37].	Not reported	Not reported
Seizures	Episodes of abnormal, excessive neuronal activity resulting in convulsions or altered neurological function.	Hall (1980) [26], Matar (2016) [49].	Not reported	Not reported

Table S7. Comprehensive Summary of Additional Developmental and Systemic Abnormalities Reported Following Prenatal Warfarin Exposure in Humans

**Phenotype**	**Description**	**Sources**	**Dosages**	
Polyhydramnios	Excess accumulation of amniotic fluid during pregnancy.	Songmen (2017) [44].	5-6 mg/day [44]	Until 35 weeks of gestation [44].
Low birth weight	Birth weight below the expected range for gestational age.	Stevenson (1980) [29], Hou (2004) [36].	5 mg/day [29, 36]	Throughout pregnancy [29, 36].
Fetal growth restriction (FGR)	Impaired fetal growth resulting in size below that expected for gestational age.	Stevenson (1980) [29], Tongsong (1999) [35], Hou (2004) [36], Mehndiratta (2010) [39], Vitale (1999) [19], Pati (1994) [46].	5 mg/day [29, 36]<br>10 mg/day [35]<br>3 mg/day [39]	Throughout pregnancy [29, 36, 35, 39].
Fetal	Postmortem degenerative	Chan (2003) [31], Barr	7.5 mg/day [45]	Discontinued one week prior

maceration	changes characterized by skin discoloration, peeling, and tissue softening following prolonged intrauterine fetal demise.	(1976) [45].		to delivery [45].
Widely spaced nipples	Increased distance between the nipples relative to thoracic width.	Harrod (1981) [32].	12.5 mg/day [32]	From the 6th through 24th week of gestation [32].
Mild hearing loss	Partial impairment of auditory function.	Harrod (1981) [32], Pauli (1987) [27].	Not reported	Not reported
Deafness	Severe or complete loss of hearing.	Raghav (2007) [37], Hall (1980) [26].	Not reported	Not reported
Hypoplastic nails	Underdevelopment of the fingernails and/or toenails.	Hou (2004) [36], Basu (2016) [42].	5 mg/day [36]	Throughout pregnancy [36].
Delayed developmental milestones	Delayed attainment of expected developmental milestones during infancy or childhood.	Raghav (2007) [37], Hall (1980) [26], Basu (2016) [42].	Not reported	Not reported
Prolonged prothrombin time (PT)	Prolongation of blood clotting time as measured by the prothrombin time assay.	Pauli (1987) [27].	Not reported	Not reported

Note: These supplementary tables provide detailed case-specific information, including reported phenotypes, maternal warfarin dose, gestational exposure period, and corresponding literature references.
